# The Biochemical Anatomy of Cortical Inhibitory Synapses

**DOI:** 10.1371/journal.pone.0039572

**Published:** 2012-06-29

**Authors:** Elizabeth A. Heller, Wenzhu Zhang, Fekrije Selimi, John C. Earnheart, Marta A. Ślimak, Julio Santos-Torres, Ines Ibañez-Tallon, Chiye Aoki, Brian T. Chait, Nathaniel Heintz

**Affiliations:** 1 Howard Hughes Medical Institute, Laboratory of Molecular Biology, The Rockefeller University, New York, New York, United States of America; 2 Laboratory for Mass Spectrometry and Gaseous Ion Chemistry, The Rockefeller University, New York, New York, United States of America; 3 CIRB, Collège de France, Paris, France; 4 Molecular Neurobiology Group, Max-Delbrück-Center for Molecular Medicine, Berlin, Germany; 5 Center for Neural Science, New York University, New York, New York, United States of America; Yale School of Medicine, United States of America

## Abstract

Classical electron microscopic studies of the mammalian brain revealed two major classes of synapses, distinguished by the presence of a large postsynaptic density (PSD) exclusively at type 1, excitatory synapses. Biochemical studies of the PSD have established the paradigm of the synapse as a complex signal-processing machine that controls synaptic plasticity. We report here the results of a proteomic analysis of type 2, inhibitory synaptic complexes isolated by affinity purification from the cerebral cortex. We show that these synaptic complexes contain a variety of neurotransmitter receptors, neural cell-scaffolding and adhesion molecules, but that they are entirely lacking in cell signaling proteins. This fundamental distinction between the functions of type 1 and type 2 synapses in the nervous system has far reaching implications for models of synaptic plasticity, rapid adaptations in neural circuits, and homeostatic mechanisms controlling the balance of excitation and inhibition in the mature brain.

## Introduction

Type 1 synapses, which were identified over fifty years ago [1], [2], mediate excitatory neurotransmission primarily through glutamate. Accordingly, the most abundant ionotropic neurotransmitter receptors present at these synapses are a-amino-3-hydroxy-5-methyl-4-isoxazolepropionic acid (AMPA) and N-methyl-D-aspartate (NMDA) type glutamate receptors, which control local flux of sodium (Na^+^), potassium (K^+^) and the second messenger calcium (Ca^++^) at the synapse. These glutamate receptors are part of a larger complex, the PSD, whose detergent insolubility makes it highly amenable to biochemical purification and analysis. Over the past several decades, a multitude of studies [3], [4], [5], [6] have identified the various components of the PSD. These include scaffolding proteins, such as PSD-95 [7], which provide a central docking station for neurotransmitter receptors and ion channels, and signaling components such as Ca^++^/calmodulin-dependent protein kinase II (CaMKII) [8], calcineurin [9] and SynGAP [10], which activate a wide variety of signal transduction pathways in response to synaptic activity. These signals both feed back onto the receptors to control synaptic strength, and transduce signals from the synapse to the interior of the cell to regulate transcription, translation and metabolism [3], [11].

Proteomic studies of type 1 synapses have revealed a surprising degree of biochemical complexity: over 1,000 different proteins have been identified as components of the PSD [5], [12], [13]. Although it is widely appreciated that many of these proteins may be present in only a subset of excitatory synapses [6], biochemical studies of the isolated NMDA receptor complex alone reveals a structure of 2–3 MDa containing 186 distinct proteins [13], [14]. Furthermore, analysis of the cerebellar Purkinje cell/parallel fiber PSD, which does not contain NMDA receptors, has revealed that most proteins within this single excitatory synapse type are involved in signal transduction [15]. The paradigm of the synapse that has emerged from these studies is that of a complex molecular machine composed of receptors and signaling molecules that can convert chemical signals arriving at the synapse into the cellular changes that underlie information processing, storage and retrieval in the nervous system [3], [11].

In contrast to the excitatory PSD, the biochemical complexity of type 2, inhibitory synapses has largely eluded neuroscientists. It is likely that the lack of information reflects both the relative rarity of type 2 synapses in the brain (they comprise only ∼5–15% of total synapse number) and the difficulty of purifying these simple structures by classical biochemical methods. Despite this, the importance of inhibitory synaptic transmission is underscored by recent studies of epilepsy, autism and schizophrenia, disorders characterized by an imbalance in inhibitory and excitatory neurotransmission [16], [17].

Type 2 synapses use γ-aminobutyric acid (GABA) or glycine as their major neurotransmitter. Activated GABA_A_ and glycine receptors control neuronal excitability through regulated Cl^-^ influx at key subcellular domains. Specificity of distinct inhibitory synapses is achieved in part through the pentameric structure of GABA_A_ receptors, which are assembled from a pool of 19 distinct subunits. These subunits confer differences in subcellular localization, pharmacological properties, and Cl^-^ conductance of the receptor [18], [19], [20]. Each subunit contains a large intracellular loop that contains sites for protein-protein interactions as well as sites for phosphorylation [21], ubiquitination [22], and palmitoylation [23], [24].

The main synaptic scaffold at type 2 synapses has been identified as gephyrin, which binds directly to both glycine and GABA receptors [25]. Although several additional GABA_A_ receptor binding proteins have been identified [26], many of these molecules are not localized specifically to synapses, and function in receptor trafficking or post-translational modification. It has also been shown in several studies that other neurotransmitter receptors, such as NMDA [27], [28], nicotinic acetylcholine (nACh) [29] and dopamine (D5) [30] receptors, can be found in the vicinity of GABAergic synapses, yet co-localization at the synapse has been demonstrated only in the case of NMDA receptors in hippocampal CA1 pyramidal neurons [27].

While it is known that posttranslational modulation of glutamate receptors occurs in response to signaling events generated at type 1 synapses and modulated by a diverse array of PSD proteins, it is unknown whether such a diversity of receptors and signaling molecules is similarly localized to type 2, inhibitory synapses. Given that the structural distinction between type 1 and type 2 synapses was reported over fifty years ago [31], and that the biochemical nature of inhibitory synapses remains unclear, we sought to determine whether type 2 synapses also function in signal processing. To this end we conducted proteomic studies of type 2 synaptic complexes isolated from mammalian cortical pyramidal neurons.

## Results

### Generation of an Inhibitory Synapse Affinity Tag

Proteomic studies of mammalian synapses have relied heavily on subcellular fractionation procedures that result in a PSD fraction enriched in synaptic proteins [12], [32], [33], [34]. While a consensus list of nearly one thousand synapse associated proteins has emerged from these studies [5], [35], [36], [37], the distribution of these proteins across specific synapse types remains unknown. Since in this study we were interested in understanding the biochemical properties of a specific class of cortical synapses, we adopted the synaptic protein profiling strategy [15], which has been developed to prepare biochemical fractions enriched in a specific synaptic complex. In this strategy, a synapse affinity tag is genetically expressed in a specific cell type of interest using bacterial artificial chromosome (BAC) transgenesis. Biochemical preparation of a synaptic fraction is followed by immunoaffinity purification and mass spectrometry of the tagged synaptic protein complex. In this way, non-synaptic proteins are depleted from the preparation, and only the proteins present at the synapse type of interest are enriched and identified by proteomic analysis.

**Figure 1:**
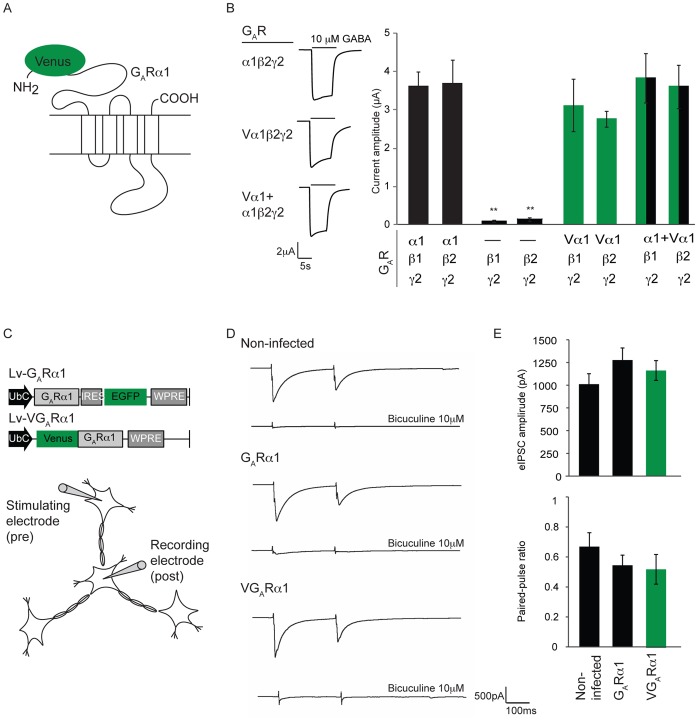
The inhibitory synapse affinity tag, Venus-GABA_A_Rα1, is functional *in vitro*. (A) Schematic of VGABA_A_Rα1, showing the N-terminal fusion of an affinity tag, Venus. (B) Representative GABA-evoked currents (left) and current amplitude quantification (right) in voltage clamped *Xenopus* oocytes after coinjection of the indicated *GABR* cRNA subunits. Values are expressed as mean ± SEM; n  = 5 oocytes per group (**p<0.01 t-test). (C) Schematic of the patch and stimulation electrodes used for paired-pulse recordings in cultured hippocampal neurons transduced with lentivirus encoding GABA_A_Rα1 (Lv-G_A_Rα1) or Venus-GABA_A_Rα1 (Lv-VG_A_Rα1) subunits. (D) Representative traces of GABAergic transmission in paired-pulse recordings in non-infected neurons and in neurons infected with the indicated lentivirus. Control traces in the presence of bicuculine are shown below each trace. (E) Quantification of the first eIPSC amplitudes and of the paired-pulse ratios obtained in the indicated neuronal cultures. Values are expressed as mean ± SEM; n  = 7−9 recorded cells per group.

For our studies of cortical inhibitory synaptic complexes by synapse protein profiling, we generated an inhibitory synapse affinity tag (VGABA_A_Rα1) by fusing an eGFP variant, Venus, to the N-terminus of the GABA_A_ receptor α1 subunit (GABA_A_Rα1) (Figure 1A) because this subunit is abundantly expressed in the cerebral cortex [19] and inserted into the majority of GABA_A_ receptors [38]. To test whether addition of the eGFP tag to the N-terminus of the GABA_A_R α1 subunit does not disrupt its ability to assemble into functional GABA_A_ receptors, comparative analysis of GABA elicited currents in *Xenopus* oocytes expressing receptors assembled from the VGABA_A_Rα1 and the wildtype GABA_A_Rα1 were performed. As shown in Figure 1B, oocytes expressing GABA_A_Rα1 in combination with the β1 or β2 and γ2 subunits show comparable current amplitudes as oocytes expressing the Venus-tagged GABA_A_Rα1 subunit, indicating that VGABA_A_Rα1 is functionally indistinguishable from the endogenous GABA_A_Rα1 subunit. As expected, in the absence of any GABA_A_Rα1 subunit, no GABA elicited currents were observed. Furthermore when oocytes expressing GABA_A_Rβ1 or β2 and GABA_A_Rγ2 were supplemented with a mixture of VGABA_A_Rα1 and wildtype GABA_A_Rα1 subunits, no change of GABA currents was detected, suggesting that the VGABA_A_Rα1 subunit does not interfere with assembly and insertion of the wildtype GABA_A_Rα1 subunit.

To ensure that overexpression of the tagged subunit VGABA_A_Rα1 does not interfere with the function of endogenous native GABA receptors, we measured evoked inhibitory post-synaptic currents (eIPSCs) by paired-pulse recordings in hippocampal neuronal cultures infected with a lentivirus driving the expression of either the wild-type GABA_A_Rα1 subunit or the VGABA_A_Rα1 subunit. Presynaptic neurons were stimulated with two consecutive short pulses and postsynaptic neurons were voltage clamped to record the amplitudes of the first and second eIPSCs (Figure 1C). In control, non-infected cultures paired-pulse stimulation produced large first postsynaptic responses and smaller second responses with low paired-pulse ratios (PPR = eIPSC2/eIPSC1<0.6) characteristic of this type of neuronal preparation [39] (Figure 1D–F). These responses were blocked by bicuculine, a selective antagonist of GABA_A_ receptors. Neuronal cultures transduced with either the wildtype or venus-tagged receptor subunits displayed comparable eIPSC amplitudes (Figure 1E) and PPR ratios (Figure 1F). Taken together, these electrophysiological data demonstrate that the VGABA_A_Rα1 subunits assemble into functional GABA_A_ receptors (oocytes) and that the function of inhibitory synapses is not altered by the presence of the eGFP affinity tag (neuronal cultures).

### Transgenic Expression of VGABA_A_Rα1

**Figure 2:**
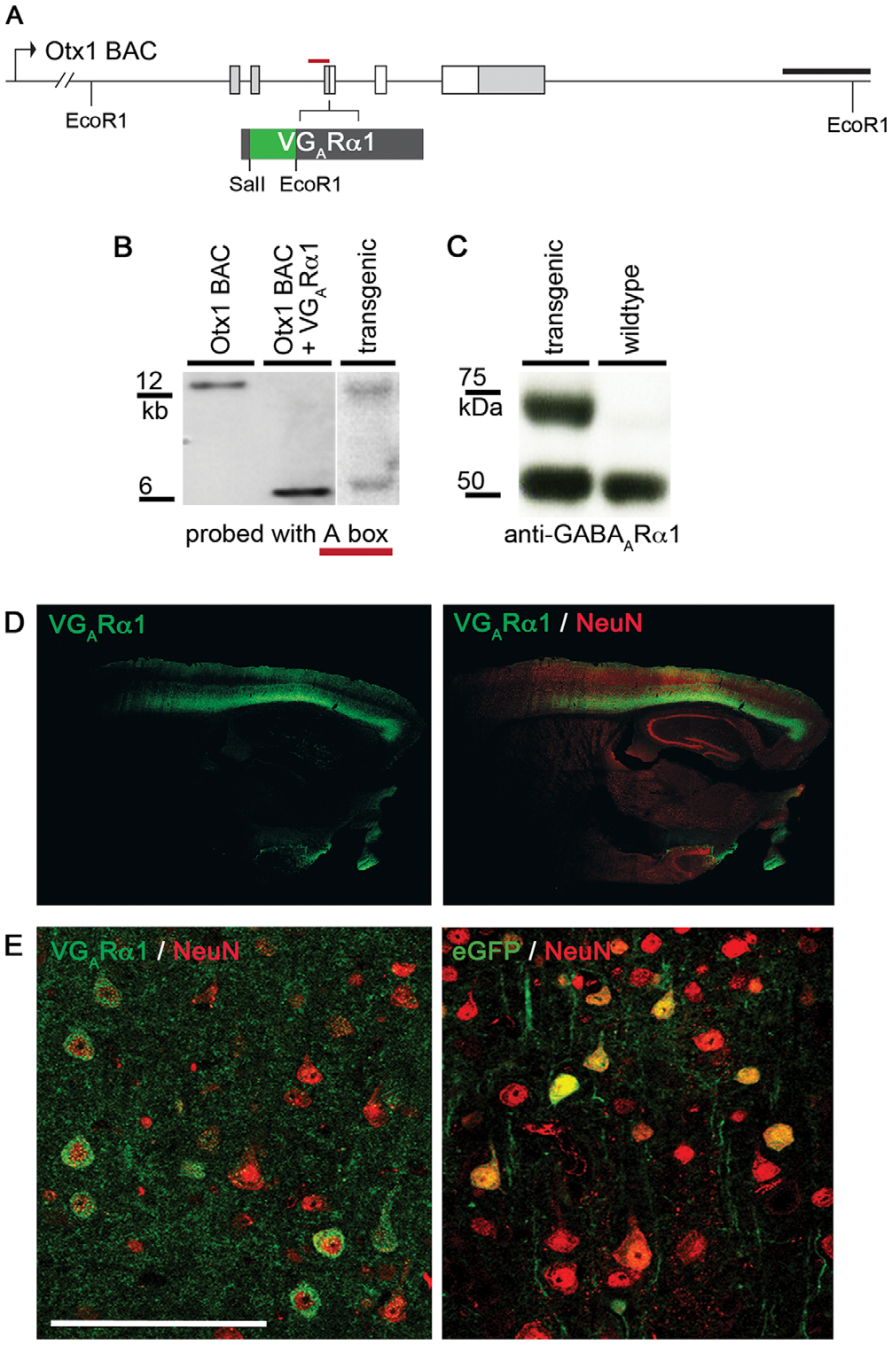
Transgenic expression of Venus-GABA_A_Rα1. (A) Strategy for Otx1 BAC modification with VGABA_A_Rα1. The red line shows the Southern blot probe used in (B). Scale: 2 kb. (B) Correct incorporation of *Venus-GABRA1* cDNA into the Otx1 BAC is shown by southern blotting. The modified BAC (middle lane) contains an additional EcoR1 site. The right lane shows correct incorporation of the modified BAC into the mouse genome. The transgenic mouse genome contains a wild-type copy of the Otx1 regulatory region as well as the modified Otx1-*Venus-GABRA1* BAC. (C) Cortical protein extract from wild type and Otx1-VGABA_A_Rα1 mice immunoblotted with anti-GABA_A_Rα1 antibody. Only the transgenic mouse expresses the fusion version of the GABA_A_Rα1 subunit (top band). (D) VGABA_A_Rα1 expression in cortical layers 5 and 6 pyramidal neurons of Otx1-VGABA_A_Rα1 mice is shown by GFP immunoreactivity. The fusion protein is localized to pyramidal cell soma in layers 5/6 and processes in layers 2/3. Scale: 500 µm. (E) Immunofluorescence shows the colocalization of VGABA_A_Rα1 (green) and NeuN (red), a neuronal marker, in layers 5 and 6 pyramidal neurons of Otx1- VGABA_A_Rα1 transgenic mice (left). VGABA_A_Rα1 is mainly localized to the perikarya of the cell soma as well as dendrites. A control Otx1 BAC transgenic mouse expresses soluble eGFP (right), which fills the cell soma. Scale: 100 µm. V: Venus. G_A_R: GABA_A_ receptor.

To tag cortical inhibitory synapses *in vivo*, we generated BAC transgenic mice expressing the VGABA_A_Rα1 subunit under the control of the Otx1 gene locus because this BAC reproducibly drives expression of transgenes in deep layer cortical pyramidal neurons [40] and transgenic mice carrying this BAC display no observable phenotype. Accordingly, VGABA_A_Rα1 was engineered into the Otx1 BAC as described previously (www.gensat.org) and Southern blot analysis used to confirm the correct modification of the Otx1 BAC (Figure 2A), and the subsequent insertion of the transgene into the mouse genome (Figure 2B). We then analyzed whole cortical protein extract for the presence of the fusion protein. Cortical extract from transgenic Otx1-VGABA_A_Rα1 mice immunoblotted with an anti-G_A_Rα1 antibody shows both the endogenous (GABA_A_Rα1) and fusion (VGABA_A_Rα1) subunits at their respective sizes, while wild-type extract contains only the endogenous subunit (Figure 2C).

To confirm correct cortical localization of the fusion protein, VGABA_A_Rα1, we performed immunofluorescence confocal microscopy on fixed brain sections of transgenic and wild type mice using an anti-GFP antibody. Low magnitude confocal images show GFP immunoreactivity in cortical neurons of transgenic mice (Figure 2D). The distribution of the fusion protein corresponds with the expected distribution of endogenous GABA_A_Rα1 subunit to cell bodies in cortical layers 5/6 and dendrites in layers 2/3, with minimal staining in layer 4 [41]. Higher magnification confocal images revealed GFP immunoreactivity for VGABA_A_Rα1 in the perikarya of layer 5/6 pyramidal neurons of transgenic mice while sections from the control Otx1-eGFP mice revealed soluble eGFP, which fills the cell soma (Figure 2E).

### VGABA_A_Rα1 Localizes to Inhibitory Synapses

**Figure 3:**
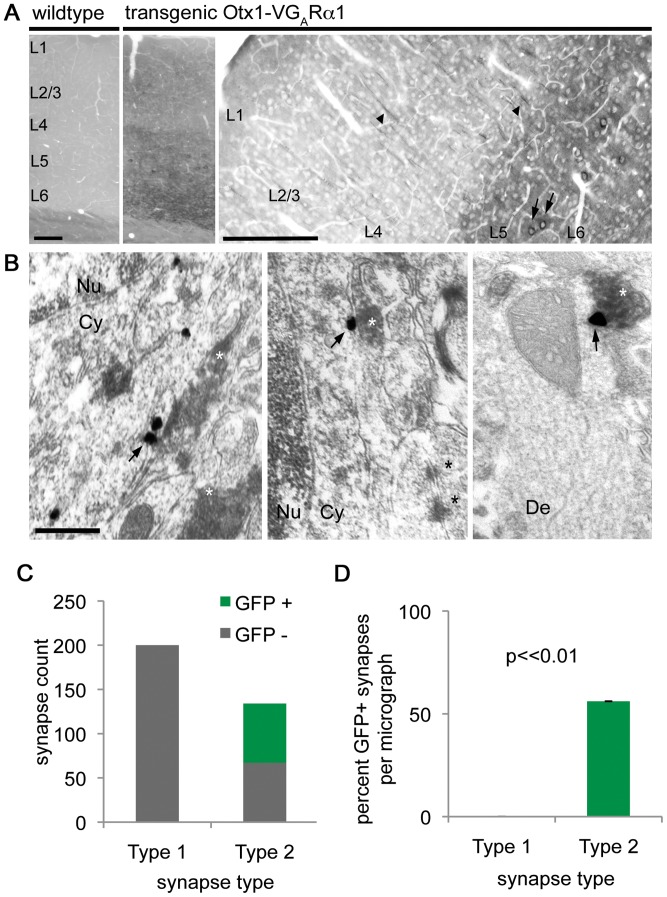
Venus-GABA_A_Rα1 localizes specifically to inhibitory synapses. (A) Light microscopy of fixed saggital sections from wild type and Otx1-VGABA_A_Rα1 transgenic mice treated with anti-GFP antibody and revealed with the DAB procedure. Transgenic, but not wild type mice express the fusion protein in layer 5/6 cortical pyramidal neurons. The fusion protein localizes to cell bodies (arrows) and processes (arrowheads) in cortex. Scale bars: 200 µm. (B) Immuno-electron microscopy shows VGABA_A_Rα1 expression (arrows) exclusively at inhibitory synapses by silver-intensified immunogold labeling (SIG). Inhibitory terminals immunoreactive for GAD65/67 are revealed with the DAB procedure (white asterisks). Asymmetric synapses (black asterisks) are immunonegative for both GAD and VGABA_A_Rα1. Scale: 500 nm. Cy: cytoplasm. Nu: nucleus. De: dendrite. V: Venus. G_A_R: GABA_A_ receptor. (C) Within a total cortical area of 614.6 square microns 67 of the 134 inhibitory (symmetric) synapses were labeled by VGABA_A_Rα1, whereas none of the 200 excitatory (asymmetric) synapses were immunopositive for the fusion protein. (D) An average of 54% of inhibitory synapses were immunopositive for VGABA_A_Rα1, compared to 0% of the excitatory synapses. The data are presented as average ± SEM (t test).

To determine whether VGABA_A_Rα1 is present at inhibitory synapses, an anti-GFP antibody was used to detect VGABA_A_Rα1 in cortical tissue. Using the DAB method to reveal GFP immunoreactivity, VGABA_A_Rα1 was present in layer 5/6 cortical pyramidal cell bodies and dendrites of transgenic but not wild type mice (Figure 3A). We then performed immuno-electron microscopy to determine the subcellular localization of our transgene and confirm its proper insertion into neuronal membranes (Figure 3B–D). Immuno-electron microscopy demonstrated that VGABA_A_Rα1 was present at the cell membrane of pyramidal neurons adjacent to axon terminals containing glutamic acid decarboxylase (GAD), a presynaptic marker of type 2 GABAergic synapses. Colocalization with GAD was seen at both the cell soma (Figure 3B, C) and dendrites (Figure 3C) of layer 5/6 pyramidal neurons.

To test whether VGABA_A_Rα1 is specifically targeted to inhibitory synapses, a total cortical area of 614.6 square microns was analyzed for the presence of VGABA_A_Rα1 at inhibitory and excitatory synapses (see experimental procedures). Within this area, 67 of the 134 inhibitory (symmetric) synapses were labeled by VGABA_A_Rα1, whereas none of the 200 excitatory (asymmetric) synapses were immunopositive for the fusion protein (Figure 3C). Within each micrograph, an average of 54% of inhibitory synapses were immunopositive for VGABA_A_Rα1, compared to 0% of the excitatory synapses (Figure 3D). These data demonstrate the specific targeting of VGABA_A_Rα1 to type 2, inhibitory synapses in layer 5/6 cortical pyramidal neurons.

### Biochemical Purification of an Inhibitory Synaptic Protein Complex

Classical excitatory synapse preparations, based on sucrose or Percoll gradients [42] have been optimized to isolate large, detergent-insoluble protein complexes typical of the excitatory PSD, and separate them from mitochondrial and reticular fractions. We have previously developed a new method for isolation of excitatory PSDs that, combined with affinity-purification, results in greater recovery and purity necessary for mass spectrometric analysis of single synapse types [15]. Inhibitory synapses are likely to contain far fewer proteins and perhaps more lipid than their excitatory counterparts given their relative structural simplicity and elongated shape [43]. Therefore, to accomplish the specific purification and enrichment of inhibitory synaptic proteins we used our new purification method modified to preserve inhibitory synaptic complexes intact but enable their affinity-purification.

The ratio of detergent to protein/lipid is a critical determinant in membrane solubilization; low ratios are necessary to purify large segments of membrane incorporating intact protein complexes, while increasing detergent will generate individual proteins in detergent micelles [44]. The selection of detergent conditions was conducted empirically, in order to maximize the recovery of intact inhibitory synaptic proteins associated with VGABA_A_Rα1. In accordance with published methods [45], we found that 0.1% beta-octylglucopyranoside (β-OG) efficiently solubilized inhibitory receptors, but failed to enrich the inhibitory synaptic complex in high molecular weight fractions via gel filtration (Figure S1C). 3-[3-cholamidopropyl-dimethylammonio]-l-propanesulfonate (CHAPS) solubilized GABA_A_ receptors [45], [46], [47], and inhibitory synaptic complexes solubilized in 0.5% CHAPS were enriched in high molecular weight fractions (Figure S1A). However, CHAPS was less efficient than Triton X-100 in removing nonspecific proteins during the subsequent affinity purification (Figure S1B).

**Figure 4:**
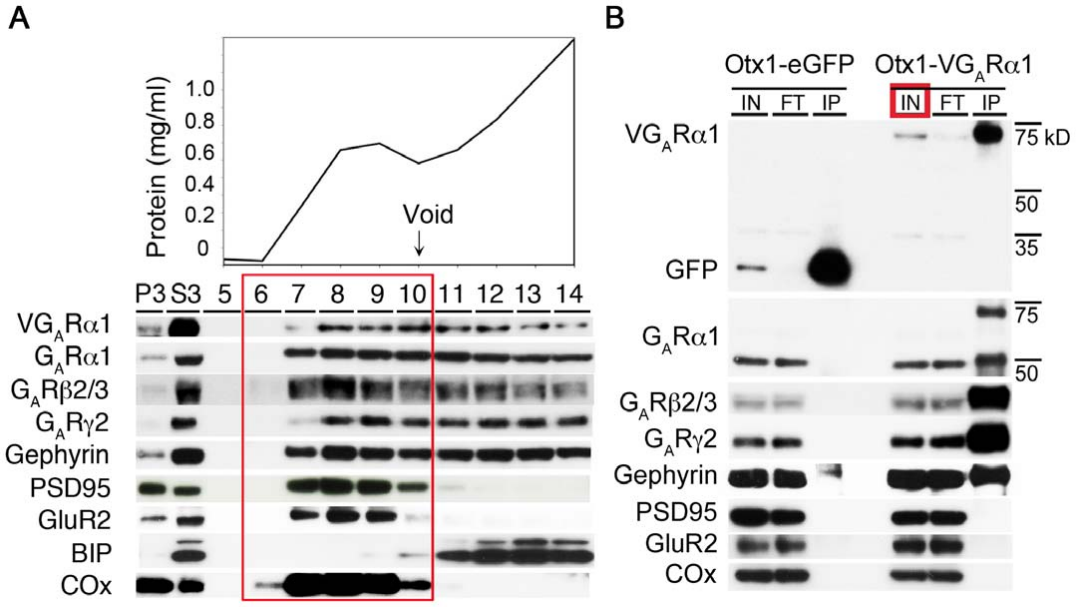
Biochemical purification of a tagged inhibitory synaptic protein complex. (A) Immunoblotting of various proteins shows that detergent solubilized protein extract S3 was enriched in both inhibitory (VGABA_A_Rα1, GABA_A_Rα1, GABA_A_Rβ2/3, GABA_A_Rγ2) and excitatory (GluR2, PSD95) synaptic proteins, as well as mitochondria (COx). Gel filtration of fraction S3 enabled enrichment of synaptic protein complexes relative to intracellular proteins, as shown by the specific exclusion of the endoplasmic reticulum marker BIP, from the high molecular weight fractions (6–10). Protein concentration of each fraction was measured (top), and the void volume determined by the elution of Blue Dextran (2000 kDa). Identical results were obtained for endogenous proteins in fractions prepared from wildtype or Otx1-eGFP cortices (not shown). (B) Fractions 6–10 (red box in A) from Otx1-VGABA_A_Rα1 or Otx1-eGFP control were pooled and subject to co-immunopurification using an anti-eGFP antibody. Immunoblotting confirmed the specific presence of inhibitory synaptic proteins (VGABA_A_Rα1, GABA_A_Rα1, GABA_A_Rβ2/3, GABA_A_Rγ2) and the absence of excitatory synaptic (GluR2, PSD95) and mitochondrial (COx) proteins in the material immunopurified via VGABA_A_Rα1. Only soluble eGFP was detected in the control sample. IN: Input. FT: Flow-through. IP: Immunoprecipitate. V: Venus. G_A_R: GABA_A_ receptor. Further biochemical experimental results are presented in Figure S1.

Triton X-100 at a concentration of 0.5% is classically used to extract excitatory PSDs and was used in our previous study of parallel fiber/Purkinje cell PSDs. Our data also showed that this concentration of Triton X-100 dissociated gephyrin from GABA_A_ receptors and thus destroyed inhibitory synaptic complexes [15]. However, we determined that mild solubilization with 0.1% Triton X-100 extracts inhibitory synaptic complexes with minimum disruption (Figures 4A, S1), Following gel filtration, high molecular weight fractions were enriched in markers of both inhibitory synapses (VGABA_A_Rα1, endogenous GABA_A_R subunits and gephyrin) and excitatory synapses, such as PSD95. Fractions eluting lower molecular weight protein complexes were enriched in the endoplasmic reticulum marker BIP and excluded from downstream affinity purification, as they contain proteins involved in neurotransmitter receptor trafficking in the interior of the cell.

Synaptic fractions were pooled (Figure 4A, red box), and subjected to affinity purification using an anti-eGFP antibody to isolate synaptic complexes containing the VGABA_A_Rα1 fusion protein (Figure 4B). To improve the specificity of our strategy, preparations were performed in parallel on Otx1 BAC transgenic mice that express soluble eGFP. In this way, proteins isolated as a result of adventitious interactions with eGFP, the beads or the antibodies could be excluded from the analysis. The choice of beads used in the subsequent immunoaffinity step of the preparation was also determined empirically (Figure S1D). We found that anti-GFP coated epoxy beads bound proteins nonspecifically, while Protein G beads did not (Figure S1). This was likely due to binding of proteins other than the monoclonal antibody to the epoxy beads, since epoxy beads coated with a gel purified monoclonal antibody immunopurified fewer nonspecific proteins (not shown). Finally, the size exclusion chromatography used in the synaptic protein profiling strategy resulted in substantially less material losses than classical methods of synapse purification which rely on centrifugation through a density gradient [15]. Taken together, these modifications to the traditional excitatory PSD preparation resulted in a procedure that is specifically tailored to isolate large, inhibitory synaptic protein complexes that are amenable to downstream proteomic analysis.

As shown in Figure 4B, the synaptic fraction purified via VGABA_A_Rα1 contained GABA_A_Rα1, β2/3 and γ2 subunits, demonstrating correct incorporation of the Venus-tagged subunit into high-molecular weight structures containing endogenous GABA_A_ receptors *in vivo*. Gephyrin, the main scaffolding protein for GABA_A_ and glycine receptors [48], [49], was also highly enriched in this fraction. As expected from the specific targeting of the VGABA_A_Rα1 affinity tag expression to a subset of deep layer pyramidal cells, only a minor fraction of the endogenous GABA_A_ receptor subunits were recovered in the affinity purification step. Notably, the primary scaffold of the excitatory PSD, PSD95, was not present in these preparations. Furthermore, no neurotransmitter receptors or scaffolding proteins (except for trace amounts of gephyrin) were recovered using the same procedures to isolate eGFP from control mice. Taken together, these data demonstrate successful enrichment of high molecular weight synaptic protein complexes from inhibitory synapses using a combination of genetically engineered transgenic mice, subcellular fractionation, and affinity purification of the VGABA_A_Rα1 synaptic tag.

### Mass Spectrometry of Cortical Inhibitory Synaptic Proteins

Studies of the synaptic proteome have typically utilized SDS-PAGE to separate complex protein mixtures and to remove detergents introduced during PSD extraction [12], [13], [35]. To avoid the material losses associated with SDS-PAGE, and to improve identification of low abundance proteins, we relied on filter-aided sample preparation (FASP) [50] a recent innovation involving microfiltration of Triton X-100 and on-filter digestion of proteins followed by liquid chromatography tandem mass spectrometry (LC-MS/MS) [51]. This departure from SDS-PAGE separation was a crucial modification to our previous strategy of synaptic protein profiling, as it facilitated retention and identification of low abundance proteins in our complex.

Proteins purified via VGABA_A_Rα1 from two replicate experiments were identified using the Global Proteome Machine (GPM) database search program X!Tandem (http://ppp.thegpm.org/tandem/ppp.html) [52] to yield a highly specific cohort of inhibitory synaptic proteins with an expectation value cutoff score of E  = 10^−4^ (Table 1, Table S1 and Figure S2). This expectation value cutoff represents the probability that the identification was random − i.e., a probability of ≤1 part in 10,000. This confidence level was independently confirmed using the target decoy strategy, which is derived from searching a database containing the sequences of all mouse proteins together with their artificially reversed sequences [53]. Although the reversed sequence database has the same size and amino acid distribution as the normal mouse protein database, it is devoid of any natural protein sequences, and provided an independent method for determining the likelihood of false positive identifications. In the present analyses, no reversed sequence proteins were detected below the E  = 10^−4^ expectation value cutoff.

**Table 1:** Inhibitory synaptic proteins identified by mass spectrometry.

			Dataset			Replicate	
Protein name	Ensembl protein ID	Mr (kD)	GPM E value	Total peptideno.	Unique peptideno.		Functional annotation
GABA_A_R β2	00000007797	54.6	10^−158^	15	7	MS/MS	Neurotransmitter receptor subunit
GABA_A_R β1	00000031122	54.1	10^−156^	15	9	MS/MS	Neurotransmitter receptor subunit
GABA_A_R β3	00000038051	54.1	10^−115^	12	5	MS/MS	Neurotransmitter receptor subunit
Neuroligin 2	00000053097	90.9	10^−111^	15	13	MS/MS	Postsynaptic cell-adhesion molecule
GABA_A_R α1	00000020707	51.7	10^−95^	13	9	MS/MS	Neurotransmitter receptor subunit
Gephyrin	00000054064	83.2	10^−86^	12	12	MS/MS	Postsynaptic scaffold protein
GABA_A_R γ2	00000063812	55.1	10^−65^	10	9	MS/MS	Neurotransmitter receptor subunit
GABA_A_R α3	00000062638	55.4	10^−45^	5	4	MS/MS	Neurotransmitter receptor subunit
Neuroligin 3	00000066304	91.1	10^−45^	5	3	MS/MS	Postsynaptic cell-adhesion molecule
Neurobeachin	00000029374	326.7	10^−40^	6	6	MS	Postsynaptic cell-adhesion molecule
GABA_A_R α2	00000000572	51.1	10^−37^	5	3	MS/MS	Neurotransmitter receptor subunit
GABA_A_R α4	00000031121	60.8	10^−37^	5	5	MS/MS	Neurotransmitter receptor subunit
GABA_A_R α5	00000063276	52.2	10^−32^	4	1	MS/MS	Neurotransmitter receptor subunit
Neurexin I	00000057294	129.6	10^−21^	4	4	MS	Presynaptic cell-adhesion molecule
GABA_A_R γ1	00000031119	53.4	10^−17^	5	4	MS	Neurotransmitter receptor subunit
LHFPL4	00000061172	27.3	10^−8^	4	4	MS/MS	Integral membrane protein
GABA_A_R δ	00000030925	50.5	10^−7^	2	2	MS	Neurotransmitter receptor subunit
Collybistin	00000085403	58.2	10^−6^	1	1	MS	Gephyrin binding protein

Proteins were purified via VGABA_A_Rα1 or eGFP and identified by LC-MS/MS. Two replicate datasets were analyzed using the GPM Database and those proteins identified in the eGFP controls were excluded from the list. Proteins present in the dataset were confirmed to be present in the replicate by either MS or MS/MS, as indicated. GPM E value is the probability that an assignment occurs by chance. Total peptide number is the total number of peptides that match a given protein. When more than one homologue is reported, unique peptide no. corresponds to the number of peptide matches that are unique to the homologue.

**Figure 5:**
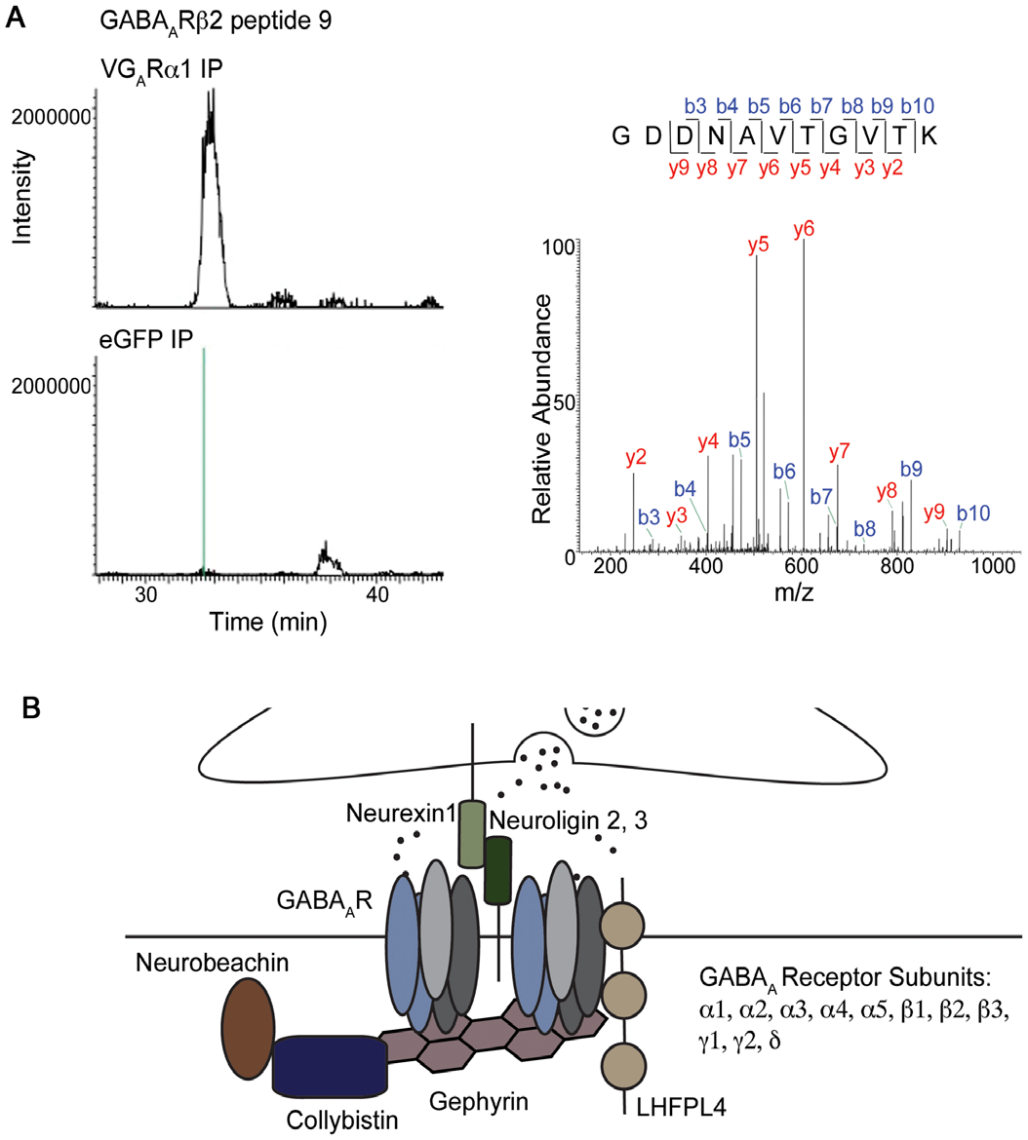
Mass spectrometry identifies proteins present at tagged inhibitory synapses. (A) All peptides were evaluated individually, for their presence or absence in the sample isolated via VGABA_A_Rα1 or eGFP, using information from peptide fragmentation spectrum (MS/MS), peptide mass spectrum (MS), and peptide retention time in extracted ion chromatogram. An example is shown for peptide, GDDNAVTGTK, from GABA_A_Rβ2. V: Venus. G_A_R: GABA_A_ receptor. (B) Schematic representation of the cortical inhibitory synaptic protein complex. These synapses contain a multitude of inhibitory receptors, as well as cell signaling and adhesion proteins, but are entirely lacking in cell signaling molecules. The localization of LHFPL4 and Neurobeachin is hypothetical. Complete information on each peptide is in Table 1 and Figure S2 and Table S1.

To confirm the specificity of the proteins identified by MS, only those proteins unequivocally identified in the VGABA_A_Rα1 pullout and not in the eGFP pullouts were considered to be specific to the inhibitory synaptic complex. For example, peptide GDDNAVTGVTK, from GABA_A_Rβ2 was present only in the material purified via VG_A_Rα1, and not found at the equivalent reversed phase HPLC retention time in the sample purified via soluble eGFP (Figure 5A). Similar mass chromatograms to those shown in Figure 5A (i.e., chromatograms showing the trace of a peptide species from a given protein with its mass specified to <10 ppm) were compared between the VGABA_A_Rα1 pullouts and controls for every peptide from every putative inhibitory synapse protein (Table S1 and Figure S2). Our analysis yielded a highly specific cohort of inhibitory synaptic proteins (Figure 5B and Table 1).

**Figure 6:**
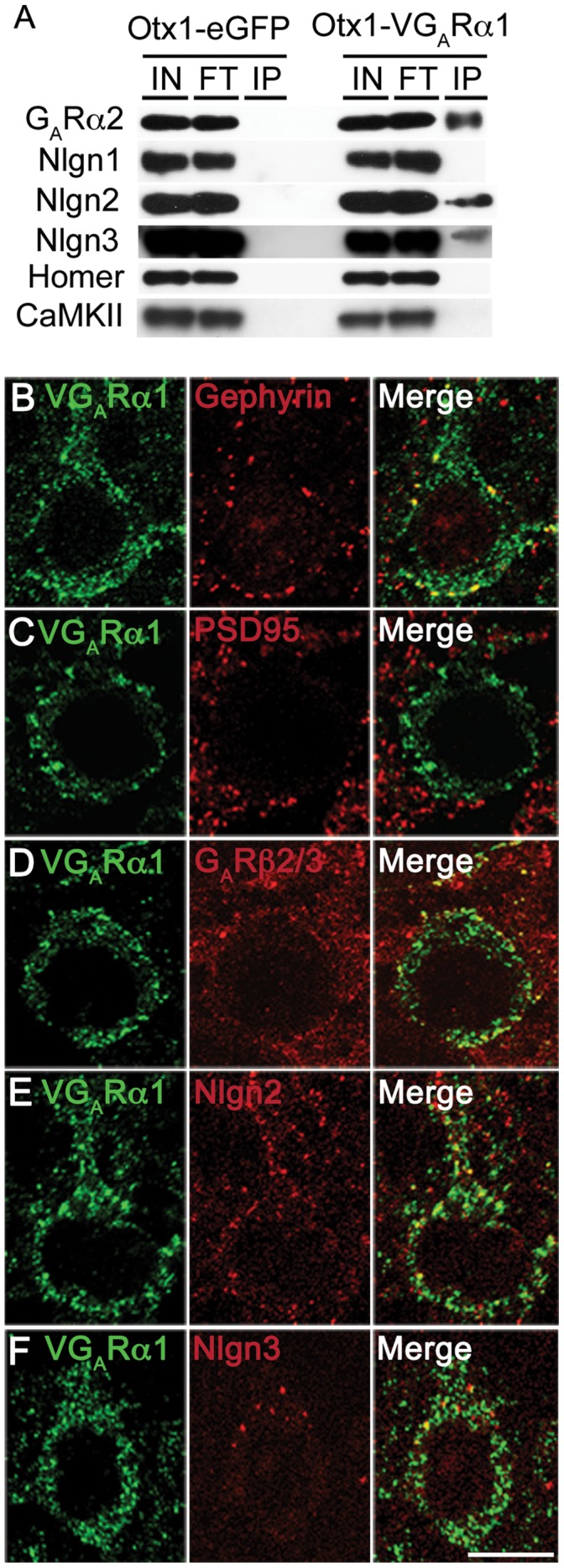
Proteins identified by mass spectrometry are present at inhibitory synapses. (A) Immunoblotting of several proteins identified by mass spectrometry confirmed their presence in immunopurified inhibitory synapses. GABA_A_Rα2, neuroligin2 and neuroligin3 are present, while excitatory markers neuroligin1 and homer are absent from VGABA_A_Rα1-tagged inhibitory synapses. The abundant signaling molecule CaMKII is also absent. (B-F) Immunofluorescence studies confirm the colocalization of several proteins identified by mass spectrometry with VGABA_A_Rα1. Gephyrin is localized to inhibitory synapses on both the cell soma and axon initial segment (B), while PSD95 is markedly absent (C). GABA_A_Rβ2/3 (D), Nlgn2 (E) and Nlgn3 (F) also colocalize with VGABA_A_Rα1 in cortical pyramidal neurons. Scale: 10 µm. V: Venus. G_A_R: GABA_A_ receptor.

The first major class of proteins isolated via tagged type 2 inhibitory synapses was GABA_A_ receptors. Eleven subunits were specifically identified, including GABA_A_R α1−5, β1−3, γ1−2, and δ. These subunits are expressed in the cerebral cortex [38], and likely comprise a mixture of high- and low-abundance receptors [19], [54]. The variety of subunits immunopurified via VGABA_A_Rα1 is consistent with the widespread distribution of the GABA_A_Rα1 subunit to the majority of cortical inhibitory synapses [38], [55]. The second major class of proteins identified consists of scaffolding and adhesion molecules. Included in this group are gephyrin, the main scaffold for inhibitory synapses, and collybistin [56], a known gephyrin binding protein. Mass spectrometry also identified two postsynaptic cell-adhesion molecules previously shown to localize to inhibitory synapses, neuroligin 2 (Nlgn2) [57] and neuroligin 3 (Nlgn3) [58]. The pre-synaptic protein neurexin 1 (Nxn1), which forms trans-synaptic adhesion complexes by binding to neuroligins, was also identified in our analysis. Finally, mass spectrometry identified two proteins whose function at synapses is less clear: neurobeachin (Nbea) and lipoma HMGIC fusion partner-like 4 (Lhfpl4). Studies of Nbea mutant mice have shown that this protein is preferentially involved in the formation and function of inhibitory synapses, and that it controls the level of expression of several proteins, including the neuroligins, at the synapse [59]. This is consistent with electron microscopic studies showing neurobeachin association with Golgi stacks and internal membranes in Purkinje neurons, and with the postsynaptic membranes of inhibitory synapses onto granule cell neurons [60]. The presence of a signal sequence and four highly conserved transmembrane domains in Lhfpl4 is also consistent with its presence in the synaptic membrane. A schematic of one possible distribution of the inhibitory protein complex is shown in Figure 5B. It is noteworthy that PSD95, CaMKII and Nlgn1, abundant scaffolding, signaling and adhesion proteins present in the synaptic fraction (Figure 4, Figure 6A) and characteristic of excitatory synapses, were absent from the affinity purified inhibitory synaptic complexes.

### Proteins Identified by Mass Spectrometry are Present at Inhibitory Synapses

The identification of Nlgns 2 and 3 in inhibitory synaptic complexes isolated from deep layer cortical pyramidal cells is of particular interest because of their association with autism spectrum disorders [17]. To confirm the mass spectrometry results, we performed western blots of affinity purified VGABA_A_Rα1 synaptic complexes (Figure 6A). Nlgns 2 and 3 were readily detected in the inhibitory synapse preparations, as was the α2 subunit of the GABA_A_ receptor. As expected, Nlgn1 and CaMKII were not detected in the inhibitory synapse preparation. Furthermore, none of these proteins were detectable in western blots of control preparations from animals expressing soluble eGFP in the same population of cortical neurons.

To examine the distribution of selected proteins identified in our analysis, we performed double immunofluorescence studies (Figure 6). Gephyrin and VGABA_A_Rα1 co-localization was seen at the perimeter of the soma and axon initial segment (Figure 6B), which reflect plasmalemmal clustering, whereas PSD95 was markedly absent from the pyramidal cell soma, and displayed a punctate staining that did not co-localize with VGABA_A_Rα1 (Figure 6C). As expected, GABA_A_R β2/3 and Nlgn2 were also evident in puncta double labeled by VGABA_A_Rα1, confirming their presence at type 2 synapses (Figures 6D, E). Nlgn3 was also present in puncta on the cell surface that co-localized with VGABA_A_Rα1 (Figure 6F), although in contrast to Nlgn2 puncta, which appeared evenly distributed over the soma in deep layer pyramidal cells, Nlgn3 puncta were present preferentially at the base of apical dendrites. In addition, Nlgn3 appeared to be much less abundant than Nlgn2, as evidenced in the immunofluorescence staining as well as our analyses by western blot (Figure 6A) and mass spectrometry (Table 1 and Figure S2). This interesting distinction between the subcellular distributions of neuroligins 2 and 3 in the cerebral cortex suggests that they may localize to distinct classes of inhibitory synapses. Indeed, previous work has demonstrated that the majority of gephyrin-immunoreactive synapses in the hippocampus contain Nlgn2, while only approximately half contain Nlgn3 [61]. Taken together, these results demonstrate that the inhibitory synapse proteomics strategy used here is sufficiently sensitive for identification of molecules present at even a small subset of type 2 synapses tagged by VGABA_A_Rα1.

## Discussion

Functional and biochemical studies of type 1 excitatory synapses have established the current paradigm of the synapse as a complex subcellular machine that upon binding of neurotransmitters to receptors located on the postsynaptic membrane, regulates the influx of Na^+^ that triggers the depolarization of that cell, and transduces local Ca^2+^ influx into a wide variety of signals that are critical for synaptic plasticity, learning and memory [3], [6], [62]. In contrast to this model, we report here that postsynaptic complexes isolated from type 2, cortical inhibitory synapses are elegantly simple: they contain neurotransmitter receptors mediating fast inhibitory neurotransmission, scaffolding molecules required to organize these receptors, and adhesion molecules that bind the postsynaptic specialization to presynaptic sites of neurotransmitter release. Despite the many signaling components identified in similar studies of type 1, asymmetric, excitatory synapses, the only protein identified in the type 2 synaptic complex preparation with domains characteristic of signal transduction proteins is the RhoGEF, collybistin. Significantly, the GDP-GTP exchange activity of collybistin is thought to participate intracellularly to regulate gephyrin clustering, not to transduce signals from the synapse to the cell interior [63]. This idea is consistent with recent studies demonstrating roles for non-synaptic GABA_A_ receptor binding proteins and other neurotransmitter receptors in the modulation of inhibitory synapses. For example, GABARAP [64], GODZ [24], and PLIC-1 [65] bind directly to GABA_A_ receptors and regulate their insertion at synapses, yet they are situated away from the postsynaptic membrane within trafficking structures such as the Golgi apparatus and ER. Although this class of proteins would not have been recovered in our study because we specifically removed trafficking complexes by size exclusion chromatography, they provide important opportunities for intracellular regulation of inhibitory synapse function. Recent studies demonstrating that activation of alpha7-nicotinic acetylcholine receptors (α7-nAchR) enhances GABAergic synaptic strength are also of interest, since they indicate that additional regulation of type 2 synapses may occur through signals from perisynaptically localized receptors [29]. Thus, our data suggest a fundamental functional distinction between excitatory and inhibitory synapses in the cerebral cortex: we propose that type 2 synapses are biochemically organized exclusively to control Cl^-^ influx at specific domains in the cell to regulate neuronal excitability.

The model of the inhibitory synapse that we propose is supported by several lines of evidence. First, recent studies of cortical synapses using focused ion beam milling and scanning electron microscopy suggest that the minimal electron density apparent at type 2 synapses may be composed only of the scaffolding proteins required to organize inhibitory receptors and adhesion molecules required to maintain synaptic contact [35], [43]. Second, we have used even gentler biochemical methods (e.g. solubilization using 0.1% instead of 0.5−1.0% Triton X-100) than those used previously to characterize the other synaptic complexes [5], [35] and have obtained high confidence scores with sensitive mass spectrometry methods to identify proteins present at affinity-tagged synapses. Perhaps the most compelling argument in support of the biochemical anatomy of the inhibitory synapse that we present here is the simple fact that Cl^-^ has not been demonstrated to serve as a second messenger for signal transduction. Thus, in contrast to the PSD of excitatory synapses, which is organized for local activation of a variety of signal transduction pathways, there is no biochemical principle for assembly of signaling proteins at the inhibitory synapse because GABA_A_ receptors do not gate the influx of a known second messenger. However, the proteomic composition of inhibitory synapses in early development is an interesting area of further study, given that GABA induced Cl^-^ flux is outward in developing neurons [66].

Consideration of the biochemical nature of the inhibitory synapse that we present here has broad implications for understanding of synaptic plasticity, neural circuit adaptation, and behavior. A cardinal prediction of this model is that plasticity of type 2 synapses will involve exclusively mechanisms that operate in the presynaptic terminal, or that are mediated by pathways triggered by intracellular signals that do originate at the synapse itself. Consistent with this idea, well characterized forms of short term plasticity at GABAergic synapses involve presynaptic mechanisms modulated by retrograde signals produced by neighboring excitatory synapses, or in response to release of Ca^2+^ from intracellular stores [67]. Although the regulation of homeostatic plasticity in inhibitory circuits is currently under investigation [68], our results suggest that the mechanisms responsible for longer term changes in inhibitory synapses will be triggered by signaling pathways distal to the synapse that regulate the synthesis, trafficking, assembly and functional characteristics of GABA_A_ receptors [69]. Given the critical role of homeostatic regulation in experience dependent plasticity, the importance of synaptic modulation at type 2 synapses for maintaining the balance of excitation and inhibition, and the relevance of these mechanisms to neurological disorders, it seems evident that the fundamental biochemical distinction between excitatory and inhibitory synapses revealed here will inform future efforts to understand detailed mechanisms of CNS function and dysfunction.

## Methods

### Detailed Experimental Procedures can be Found in Text S1

#### Animals

The cDNA encoding *GABRA1* together with the 3′UTR was amplified from cortical RNA, and placed in frame with a preprotrypsin signal sequence and Venus prior to insertion into the Otx1 BAC [40]. BAC transgenic mice were bred on the FVB background and littermates were used as wild-type controls. All experiments using animals were performed according to protocols approved by the Institutional Animal Care and Use Committee at The Rockefeller University.

### Two-Electrode Voltage-Clamp Recordings of Xenopus Oocytes

cDNA encoding for the *Venus*-*GABRA1* subunit was subcloned into the pCS2A plasmid for oocyte expression. cDNA clones of the rat subunits *GABRA1, GABRB1, GABRB2, GABRG2* in pGEMHE plasmid were a generous gift from Dr. Myles Akabas (Albert Einstein Institute, New York). All clones were in vitro transcribed with T7 or SP6 RNA polymerases (mMESSAGE mMACHINE, Ambion, Austin, TX) as described [70]. Oocytes were surgically removed and prepared as described [71]. Each oocyte was injected with 20 nl of a cRNA mix containing 1 ng of each subunit. Macroscopic currents were recorded 2 days after injection with a GeneClamp 500 B amplifier (Axon Instruments) using a two-electrode voltage clamp with active ground configuration.

### Lentivirus Production and Paired-Pulse Recordings in Primary Neuronal Cultures

Recombinant lentiviral vectors were prepared using transient transfection of HEK293T cells as described [39]. Dissociated hippocampal cultures were prepared from embryonic day 19 rat embryos and prepared as described [39]. The influence of VGABA_A_Rα1 subunit on synaptic activity was analyzed by quantification of evoked inhibitory postsynaptic currents (eIPSCs) with paired-pulse stimulation in hippocampal neuron cultures as previous reported [39]. The bath solution contained: 105 mM NaCl, 3 mM KCl, 10 mM HEPES, 5 mM glucose, 2 mM CaCl_2_ and 1 mM MgCl_2_. The recording pipette solution contained: 3 mM NaCl, 90 mM KCl, 5 mM EGTA, 5 mM HEPES, 5 mM glucose, 0.5 mM CaCl_2_ and 4 mM MgCl_2_. eIPSCs were isolated by blocking glutamatergic input (CNQX, 10 µM; DL-APV, 50 µM). Inverted eIPSCs were induced with a stimulation pipette similar to the recording pipette in close proximity to presynaptic neurons. To confirm recorded eIPSCs, bicuculline was to block the evoked GABAergic currents. The stimulation rate ensured recovery of presynaptic terminals from previous stimulation (intersweep interval 30 s). Data acquisition and analysis were performed with software Patchmaster and Fitmaster (HEKA Electronics), and with Mini Analysis program (Synaptosoft Inc).

### Immunofluorescence and Confocal Laser Scanning Microscopy

For images taken at lower magnification sections were prepared according to previously published protocols [72]. Mice were transcardially perfused with 4% paraformaldehyde in PBS, postfixed, and equilibrated in 30% sucrose. Serial free-floating coronal sections (35 µm) through the cortex were processed for immunofluorescence. For images taken at higher magnification, mice were euthanized by CO_2_ asphyxiation and fresh frozen sections (14 µm) were prepared using a cryostat. Slide-mounted sections were fixed in 4% paraformaldehyde and double or triple immunofluorescence staining was performed [73]. The sections were analyzed by confocal laser scanning microscopy (Zeiss LSM 510) using Image J software (NIH).

### Immuno-Electron Microscopy

Double immuno-electron microscopy was performed to determine whether VG_A_Rα1 occur postsynaptic to GABAergic axon terminals, latter of which were identified by the presence of the GABA-synthesizing enzyme, glutamic acid decarboxylase (GAD). Double labeling used 3,3-diaminobenzidine HCl (DAB) and silver-intensified colloidal gold (SIG) as immunolabels [74]. Brains of WT and Otx1-VG_A_Rα1 transgenic mice were fixed by transcardial perfusion of 0.1% glutaraldehyde, mixed with 4.0% paraformaldehyde in 0.1 M phosphate buffer (PB, pH 7.4). Layer 5/6 pyramidal neurons were identified by both position and immunoreactivity for the transgene. Images used for the Figure were captured digitally using a Hamamatsu CCD camera attached to a JEOL 1200XL electron microscope at a magnification of 40,000x and spanning an area of 29 mm^2^.

### Preparation of Synaptic Protein Complexes and Affinity Purification

Five cortices from adult mice were used for the preparation of a crude synaptosome fraction based on previously published protocols [42]. The solubilized fraction was separated by gravity flow on a gel-filtration column (Sephacryl S1000 Superfine, GE Healthcare). Pooled fractions from the column were used for affinity-purification of tagged inhibitory synaptic protein complexes using anti-GFP conjugated Dynabeads Protein G beads (Dynal, Oslo).

### Mass Spectrometry

Mass spectrometry analysis was performed on proteins affinity isolated via VG_A_Rα1 (tagged sample) or eGFP (control sample) and digested using the “FASP II” on-membrane digestion protocol [50] using an LTQ-Orbitrap XL mass spectrometer. Protein identification was carried out by searching a mouse protein sequence database using The Global Proteome Machine (GPM) database search program X!Tandem (http://ppp.thegpm.org/tandem/ppp.html) [52]. Proteins that were uniquely affinity isolated via VG_A_Rα1 were then determined by subtracting proteins that were immunoisolated via eGFP.

### Statistical Analysis

Unpaired two-tailed Student’s t tests were used for analyzing the data. Results are presented as means ± SEM.

## Supporting Information

Figure S1 **Biochemical enrichment of an inhibitory synaptic protein complex.** (A) CHAPS solubilize intact inhibitory synapses, as shown by enrichment of inhibitory synaptic proteins in high molecular weight fractions (6–10) following size exclusion chromatography. (B) 0.5% CHAPS is less efficient than Triton X-100 in clearing contaminant proteins during an affinity purification step. Input material is from Otx1-eGFP cortices fractions 6–10. (C) Solubilization of cortical synapses with 1% β-octylglucoside (β-OG) disrupts inhibitory synaptic protein complexes. Inhibitory GABA receptor subunits elute in low-molecular weight fractions. (D) Epoxy-coated magnetic beads bind nonspecific proteins during an affinity purification step, compared to Protein G coated beads. Beads were coupled to a monoclonal anti-eGFP antibody. Affinity purification from control Otx1-GFP mice using epoxy-beads resulted in contaminating proteins present in the eluate, which included PSD95, GABA_A_Rα1 and β2/3.
(TIF)

Figure S2 **Mass spectrometry identifies proteins present at tagged inhibitory synapses.** (A-Q) All peptides were evaluated individually, for their presence or absence in the sample isolated via VGABA_A_Rα1 or eGFP, using information from peptide fragmentation spectrum (MS/MS), peptide mass spectrum (MS – not shown), and peptide retention time in extracted ion chromatogram. An example peptide is shown for each protein listed in Table 1. For cases in which MS/MS data was only available for one of the two data sets (VGABA_A_Rα1 or VGABA_A_Rα1 Replicate), both chromatograms are shown. In cases in which the control chromatogram (eGFP IP) contained a peak at the equivalent retention time, the corresponding MS (not shown) was analyzed to determine whether the peak contained the equivalent or background peptide.
(PDF)

Table S1 **Detailed analysis of peptides identified by LC-MS/MS.** (A) Proteins were identified by the GPM protein sequence database search program X!Tandem using data from the LC/MS/MS experiments. Ensemble ID is the protein accession number in the Ensemble Mouse database. Paralogues sharing some of the indicated peptides are given in brackets. (B) Peptide sequences observed in the LC/MS/MS experiment. The symbol “*” indicates peptide sequences that also appear in protein paralogues. In cases where the peptides are modified, italic *Q* stands for Pyroglutamate formed at N-terminal Gln, italic *M* for oxidized Met, italic *W* for oxidized Trp and italic *A* for acetylated N terminal Ala. Representative mass chromatograms and MS/MS data for bold format peptides are show in Figure 4 and Figure S3. (C) Experimental peptide molecular masses. (D) Experimental peptide molecular masses – calculated peptide molecular masses. (E) Charge state of the observed peptide ions. (F) Mass to charge ratio of the observed peptide ions. (G) Experimental evidence for peptides’ presence in VGABA_A_Rα1 sample. MSMS stands for the peptide being identified via its fragmentation spectrum. MS stands for evidence of the presence of a peptide via its accurately measured mass. (H) Experimental evidence for peptides’ presence in replicate VGABA_A_Rα1 sample and GFP control sample. The symbol ‘-’ indicates non-observation of the peptide.
(PDF)

Text S1 **Detailed experimental procedures and supporting information references.**
(DOCX)
