# Supplementary figures and images for: The Biochemical Anatomy of Cortical Inhibitory Synapses
Source: PLoS One. 2012 Jun 29;7(6):e39572. doi: 10.1371/journal.pone.0039572 (PMC3387162; doi:10.1371/journal.pone.0039572)

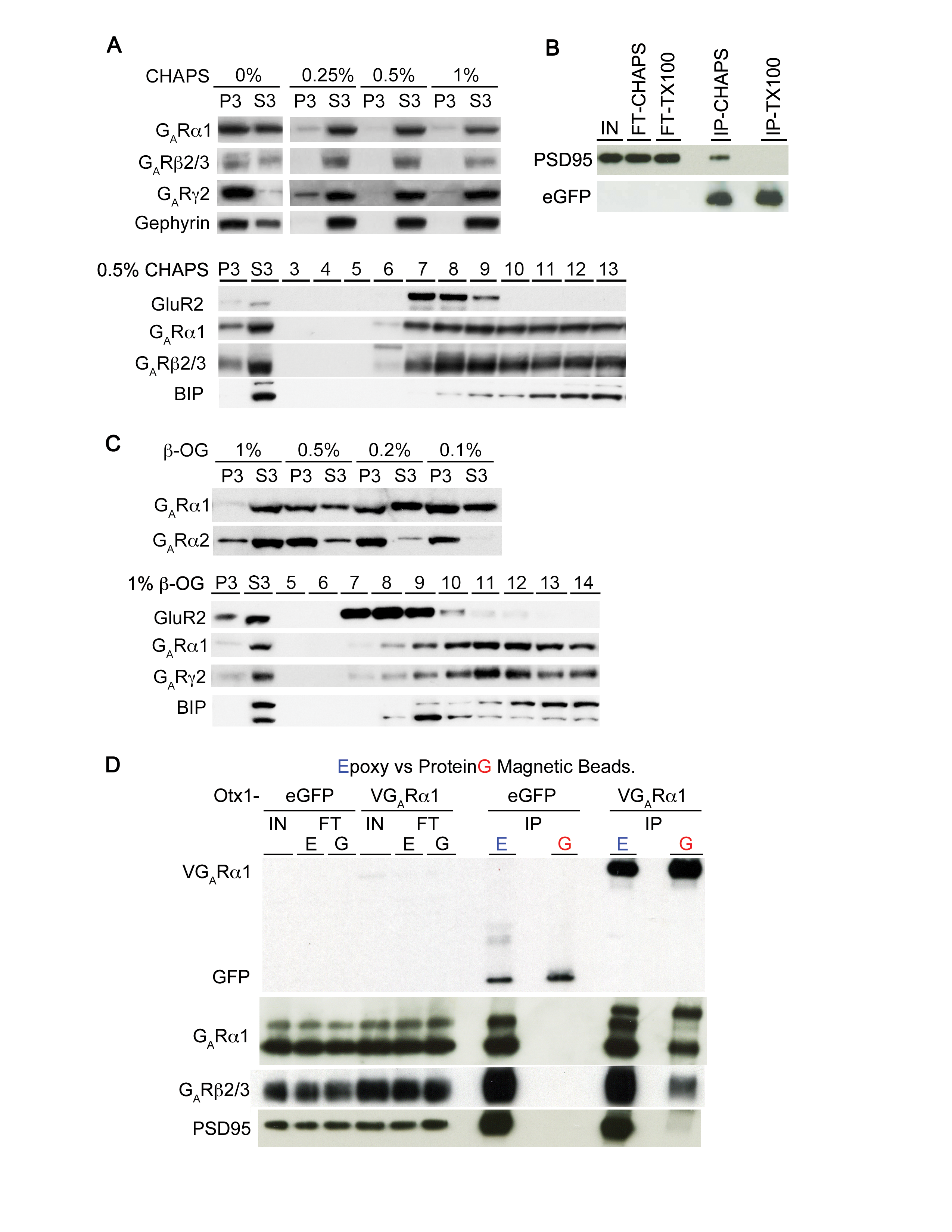

Supplement: Figure S1 — Biochemical enrichment of an inhibitory synaptic protein complex. (A) CHAPS solubilize intact inhibitory synapses, as shown by enrichment of inhibitory synaptic proteins in high molecular weight fractions (6–10) following size exclusion chromatography. (B) 0.5% CHAPS is less efficient than Triton X-100 in clearing contaminant proteins during an affinity purification step. Input material is from Otx1-eGFP cortices fractions 6–10. (C) Solubilization of cortical synapses with 1% β-octylglucoside (β-OG) disrupts inhibitory synaptic protein complexes. Inhibitory GABA receptor subunits elute in low-molecular weight fractions. (D) Epoxy-coated magnetic beads bind nonspecific proteins during an affinity purification step, compared to Protein G coated beads. Beads were coupled to a monoclonal anti-eGFP antibody. Affinity purification from control Otx1-GFP mice using epoxy-beads resulted in contaminating proteins present in the eluate, which included PSD95, GABAARα1 and β2/3. (TIF) [file pone.0039572.s001.tif]
