# Supplementary material for: The Biochemical Anatomy of Cortical Inhibitory Synapses
Source: PLoS One. 2012 Jun 29;7(6):e39572. doi: 10.1371/journal.pone.0039572 (PMC3387162; doi:10.1371/journal.pone.0039572)

FIGURE S2

A $GABA_AR\beta1$ peptide 1

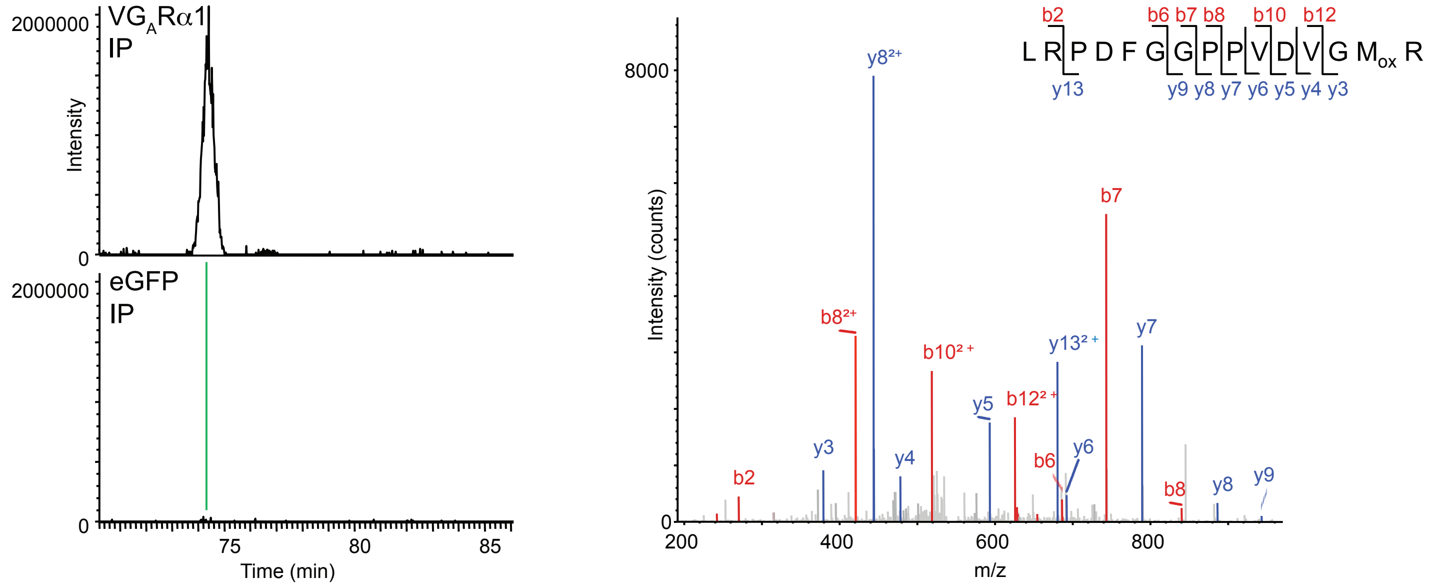

B $GABA_AR\beta3$ peptide 12

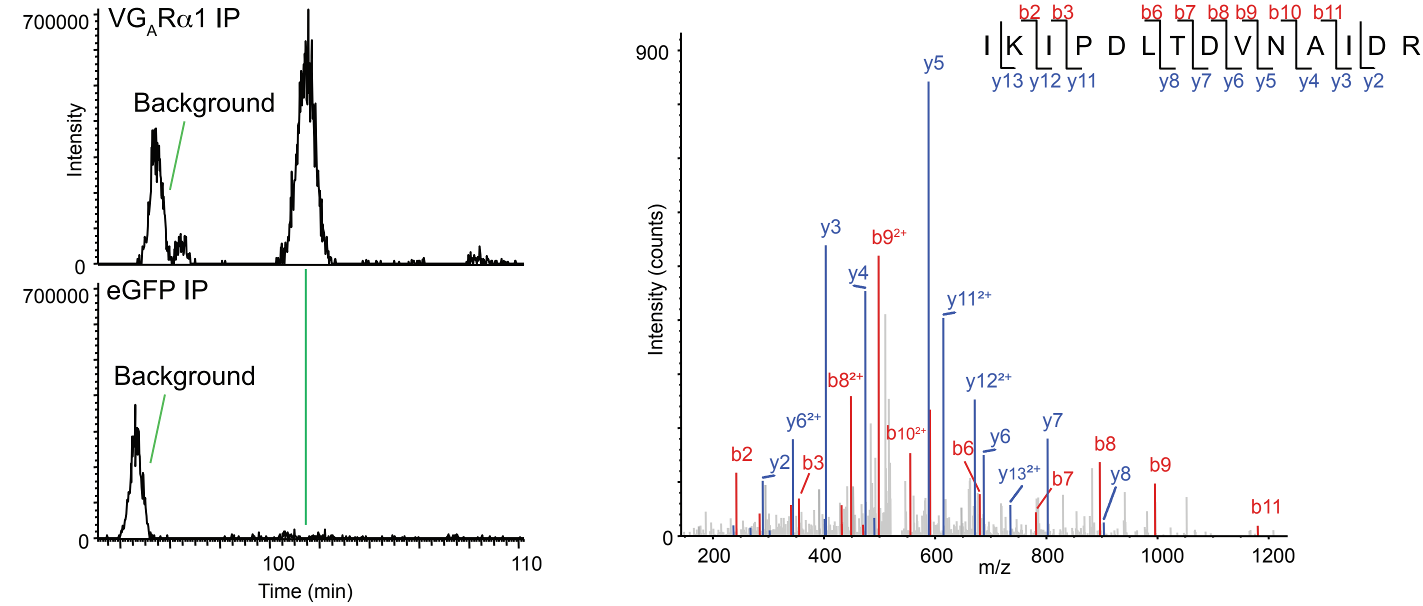

C Neuroligin2 peptide 11

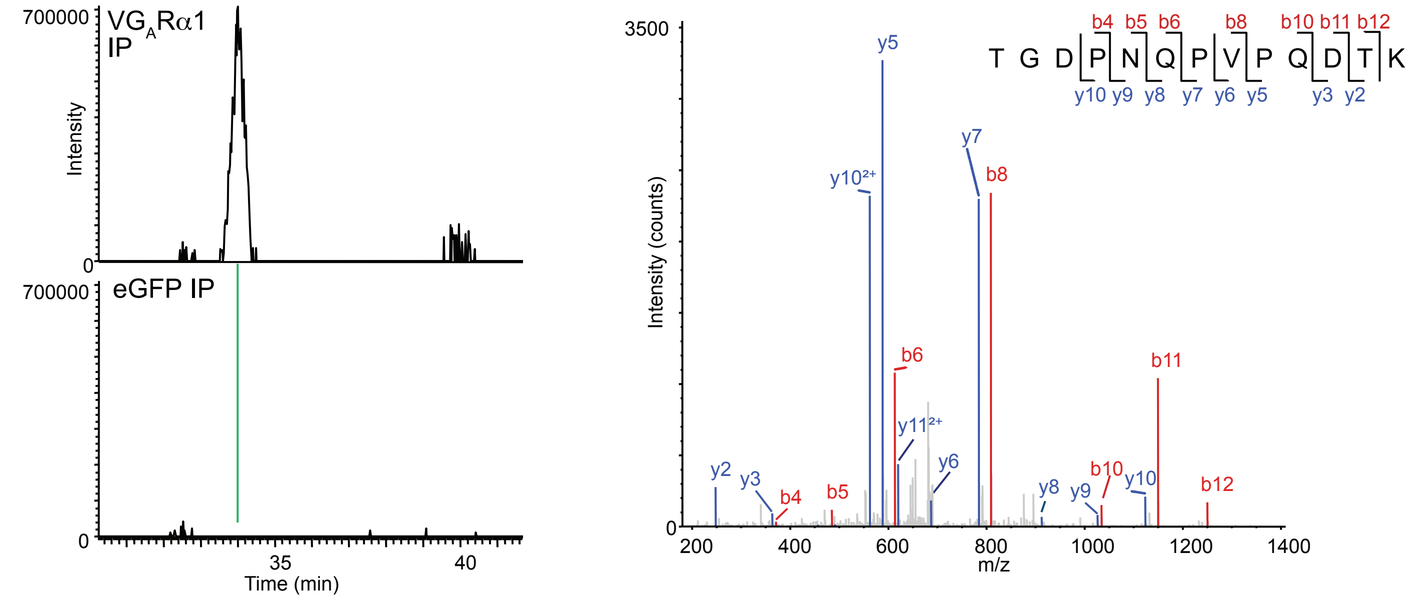

D GABA$_A$Rα1 peptide 9

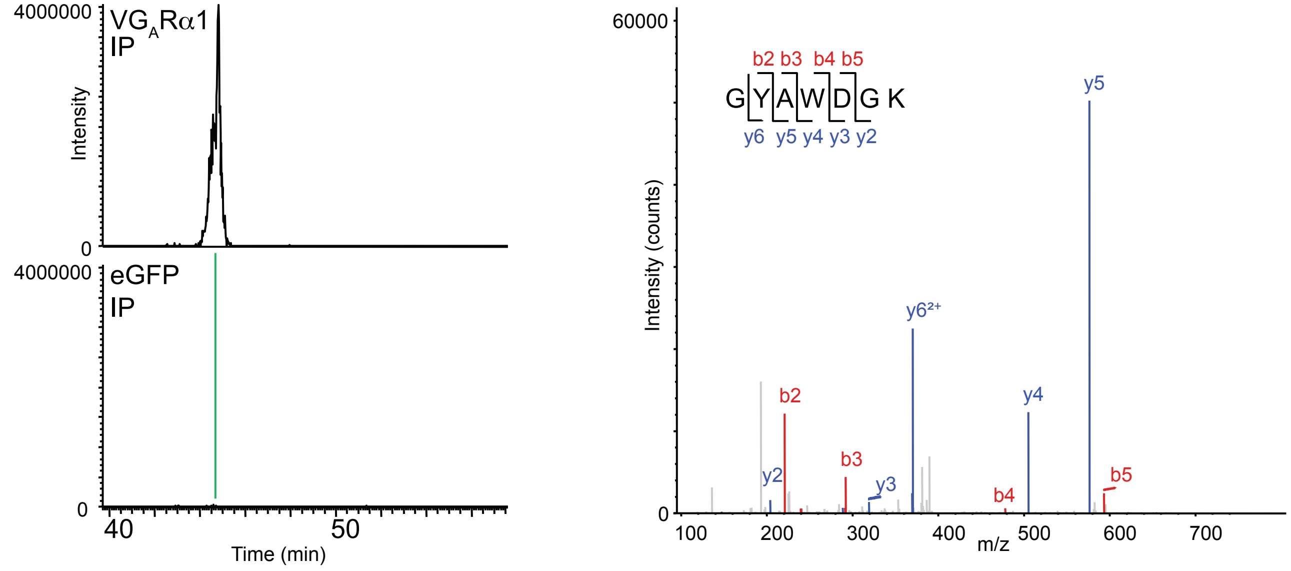

E Gephyrin peptide 12

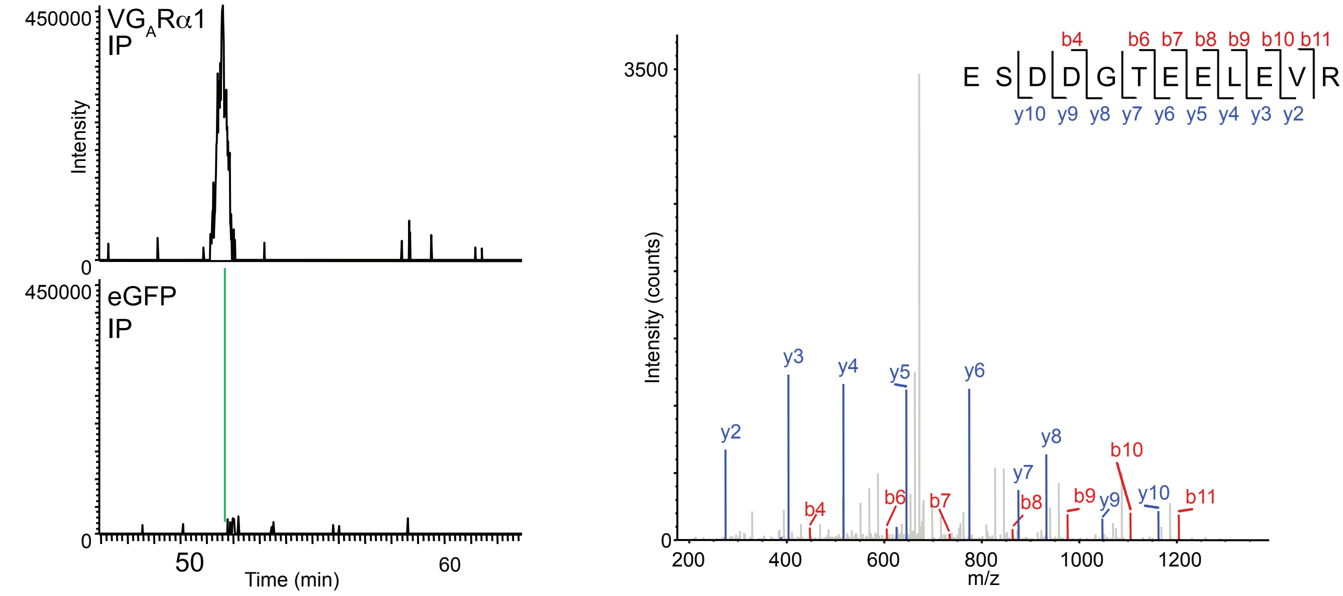

F GABA$_A$Rγ2 peptide 10

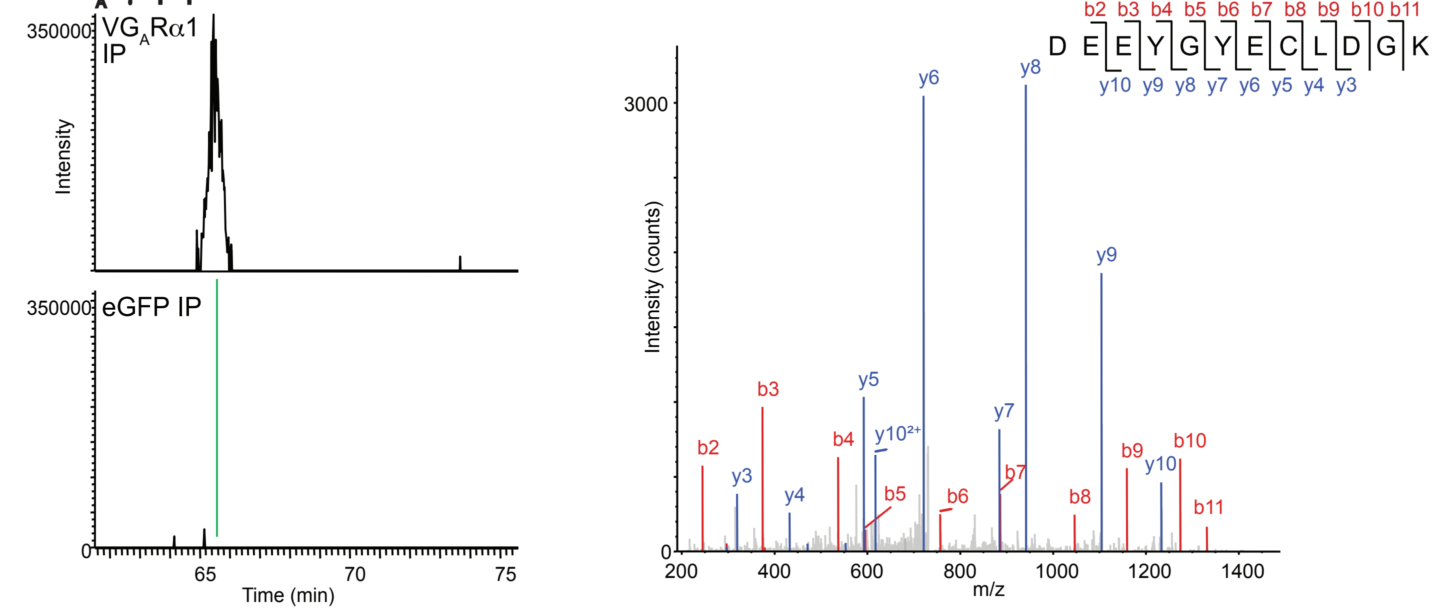

G GABA$_A$Rα3 peptide 1

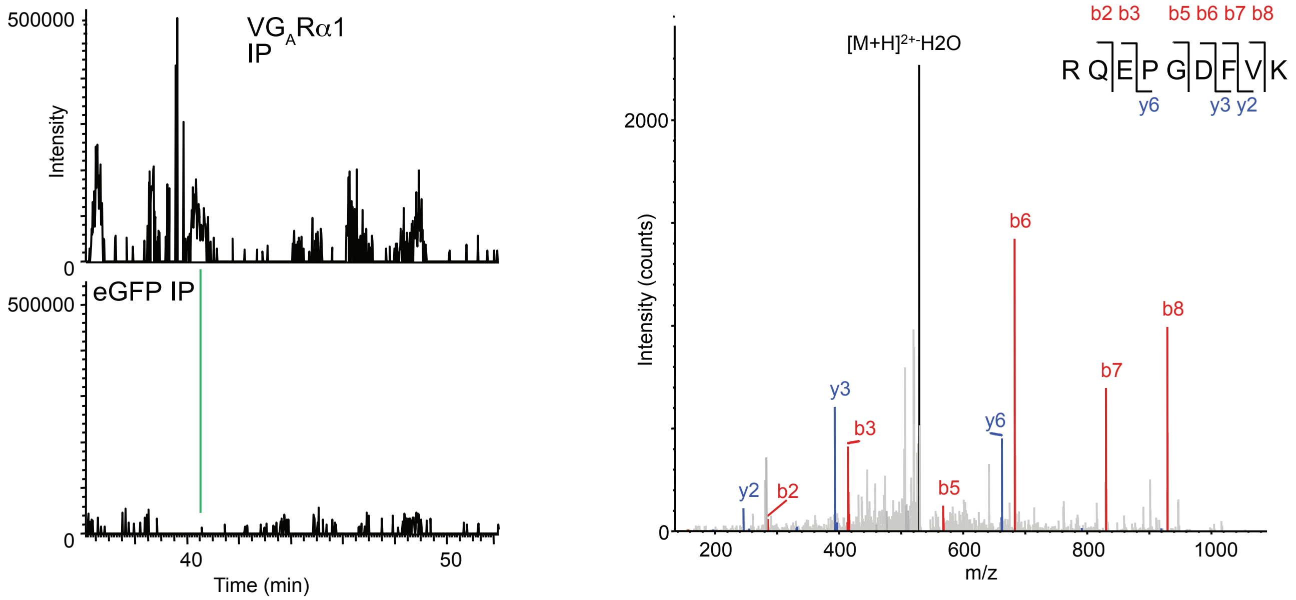

H Neuroligin3 peptide 5

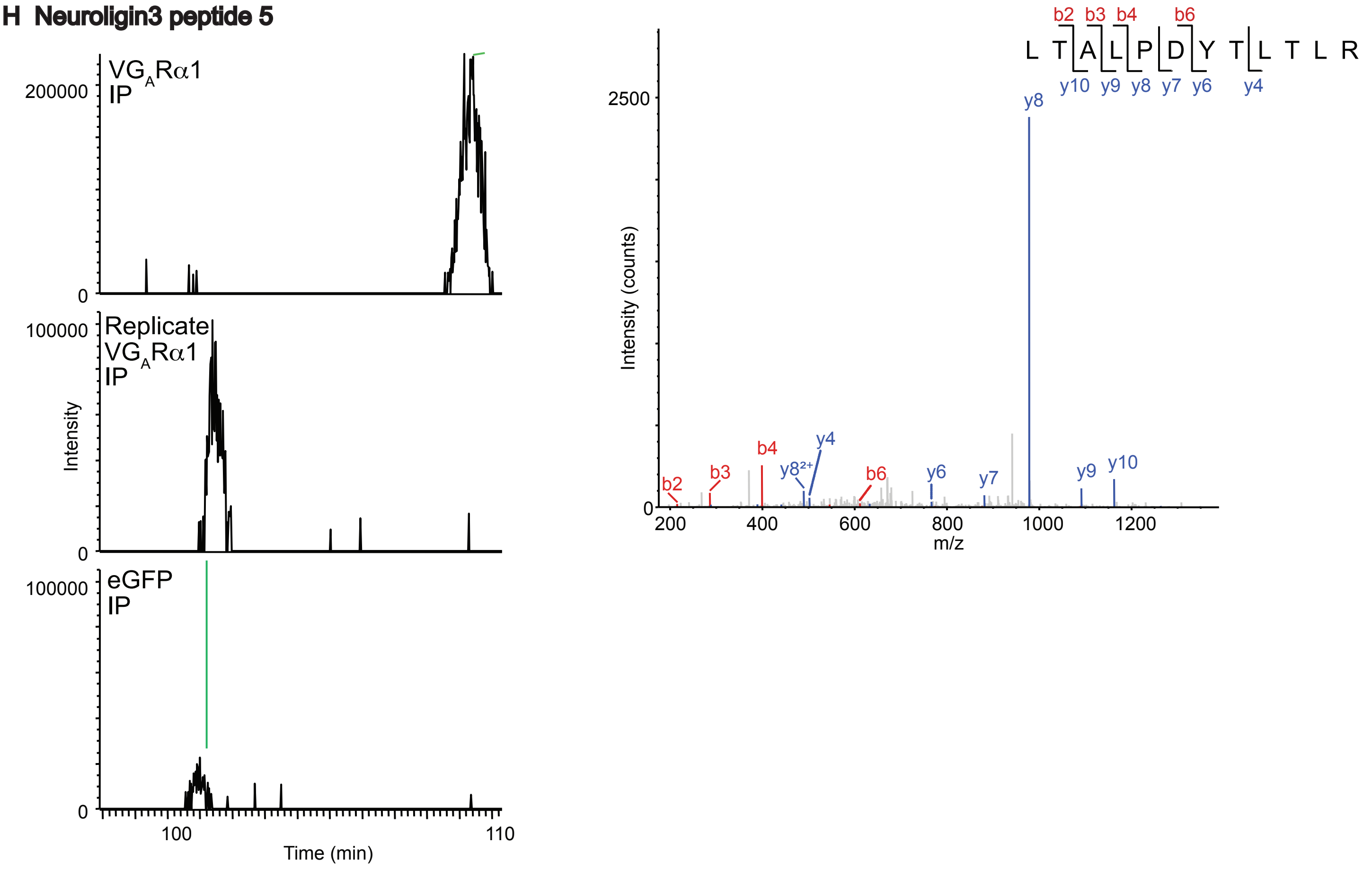

I Neurobeachin peptide 5

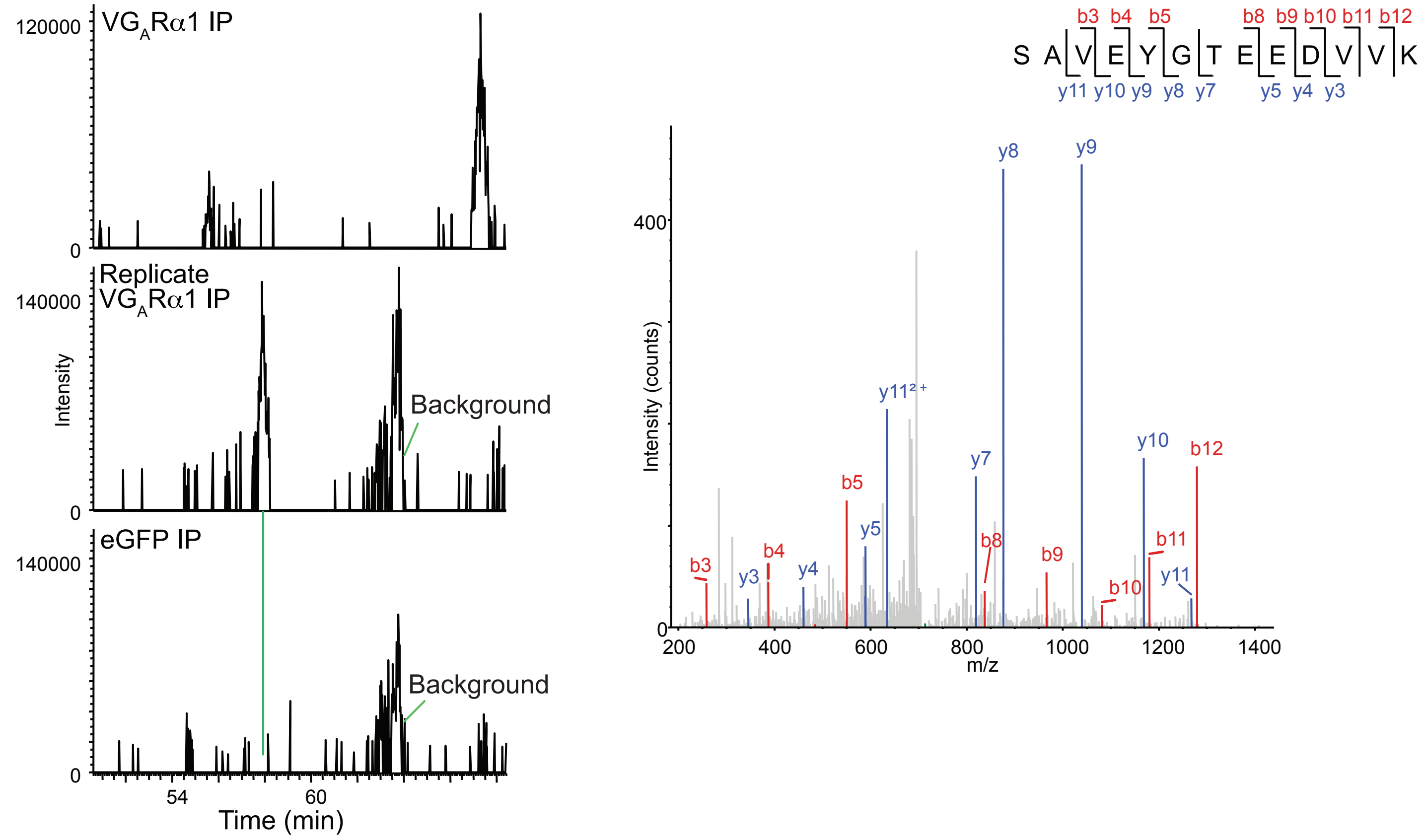

J GABA$_A$Rα2 peptide 4

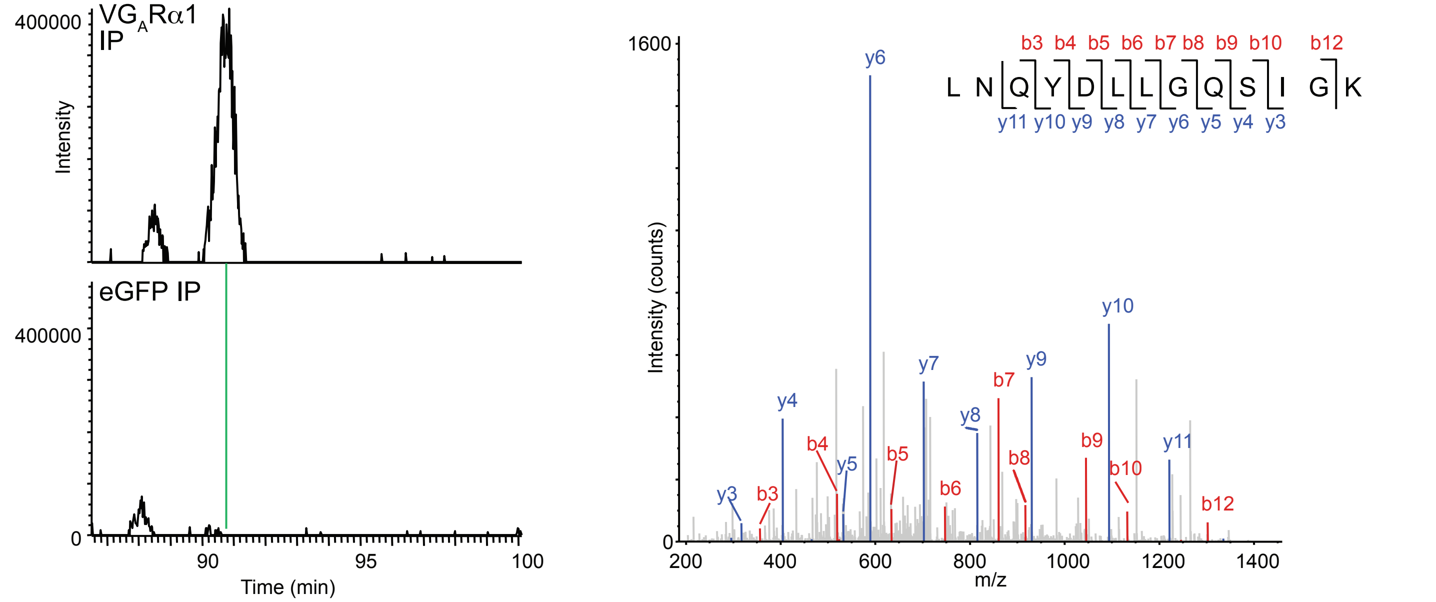

K GABA$_A$Rα4 peptide 1

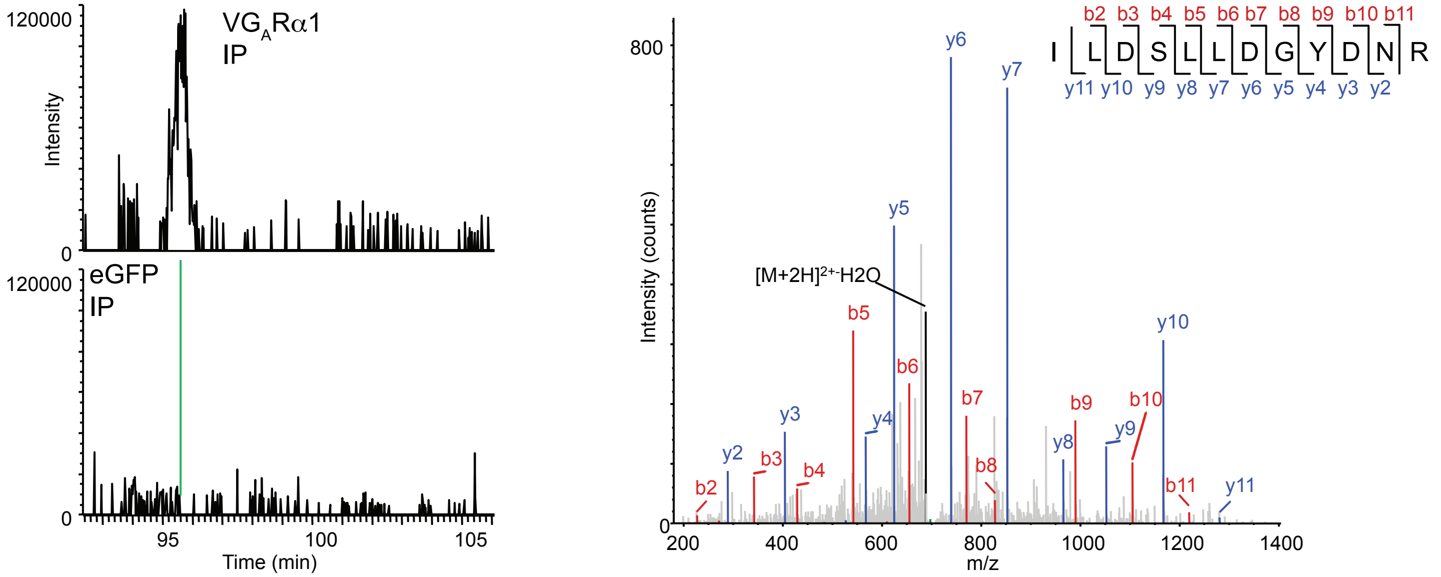

L GABA$_A$Rα5 peptide 1

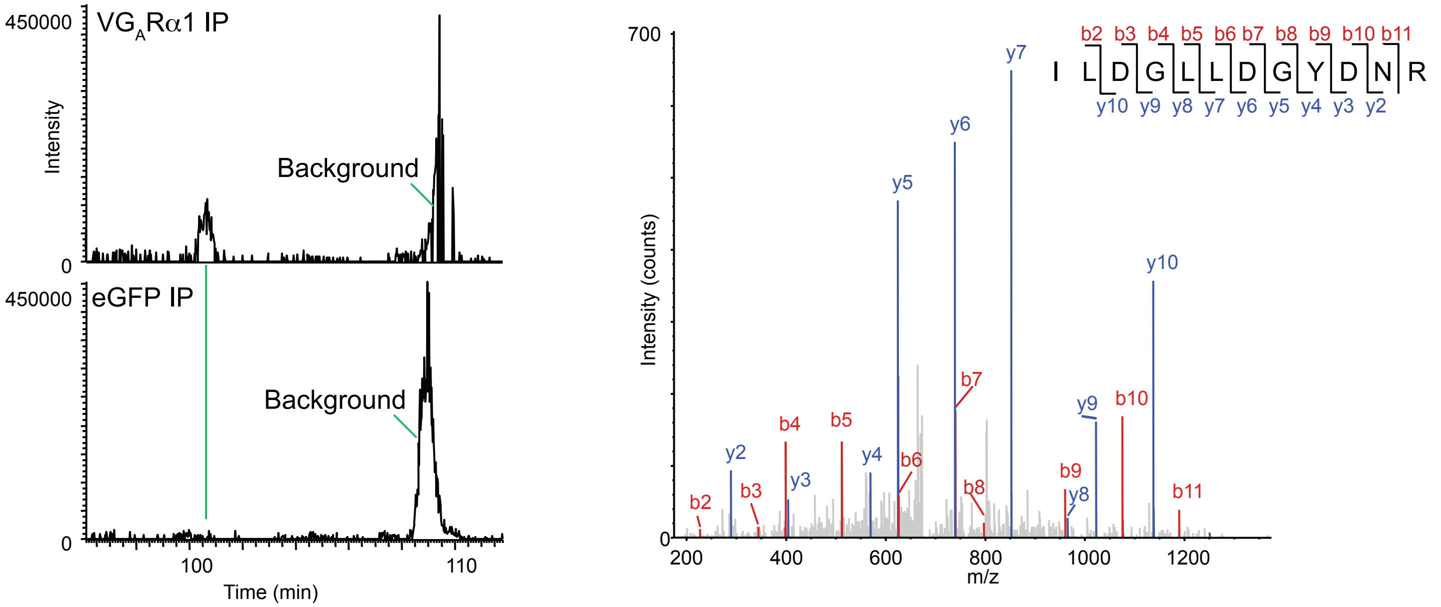

M Neurexinl peptide 5

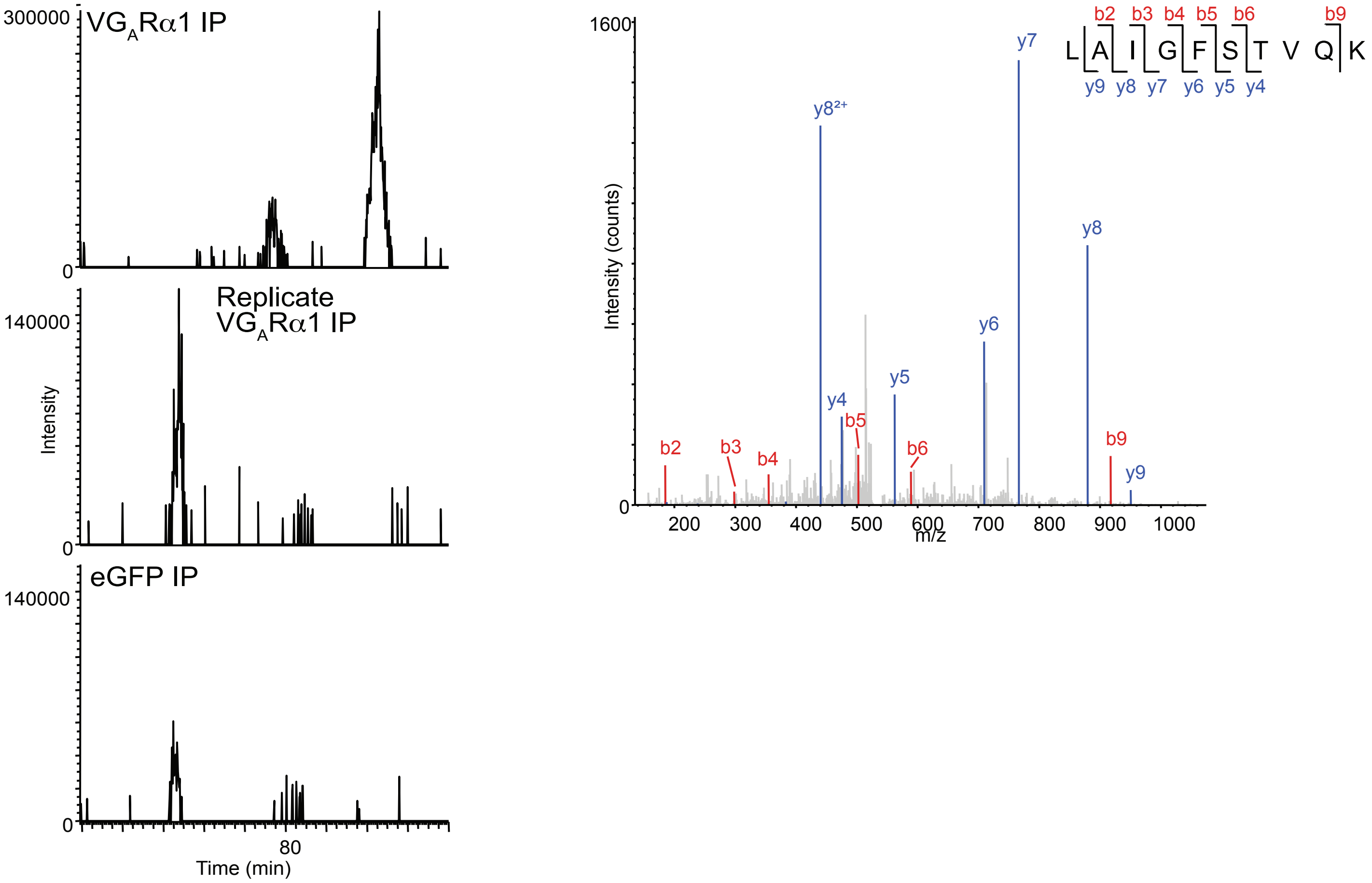

N GABA$_A$Rγ1 peptide 4

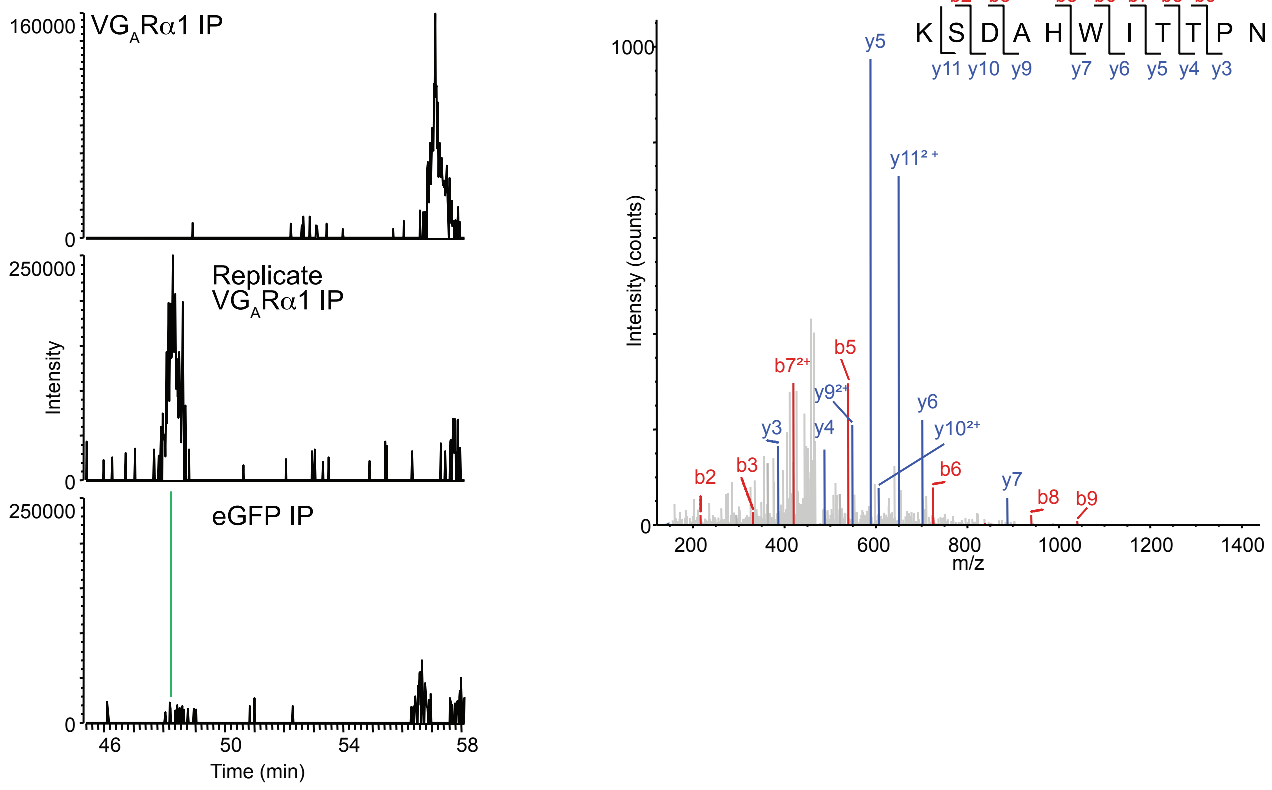

O Lhfpl4 peptide 2

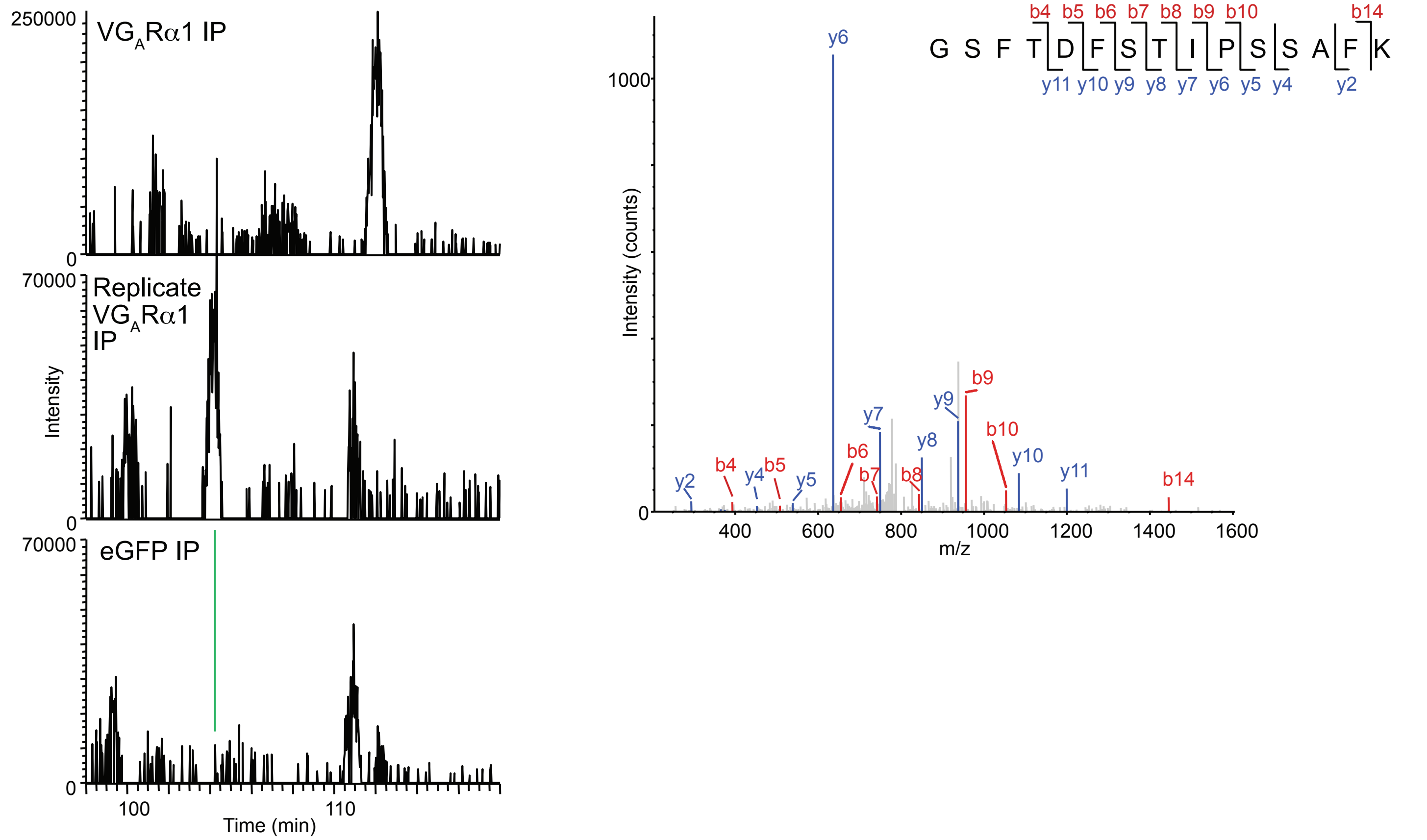

P GABA$_A$Rδ peptide 2

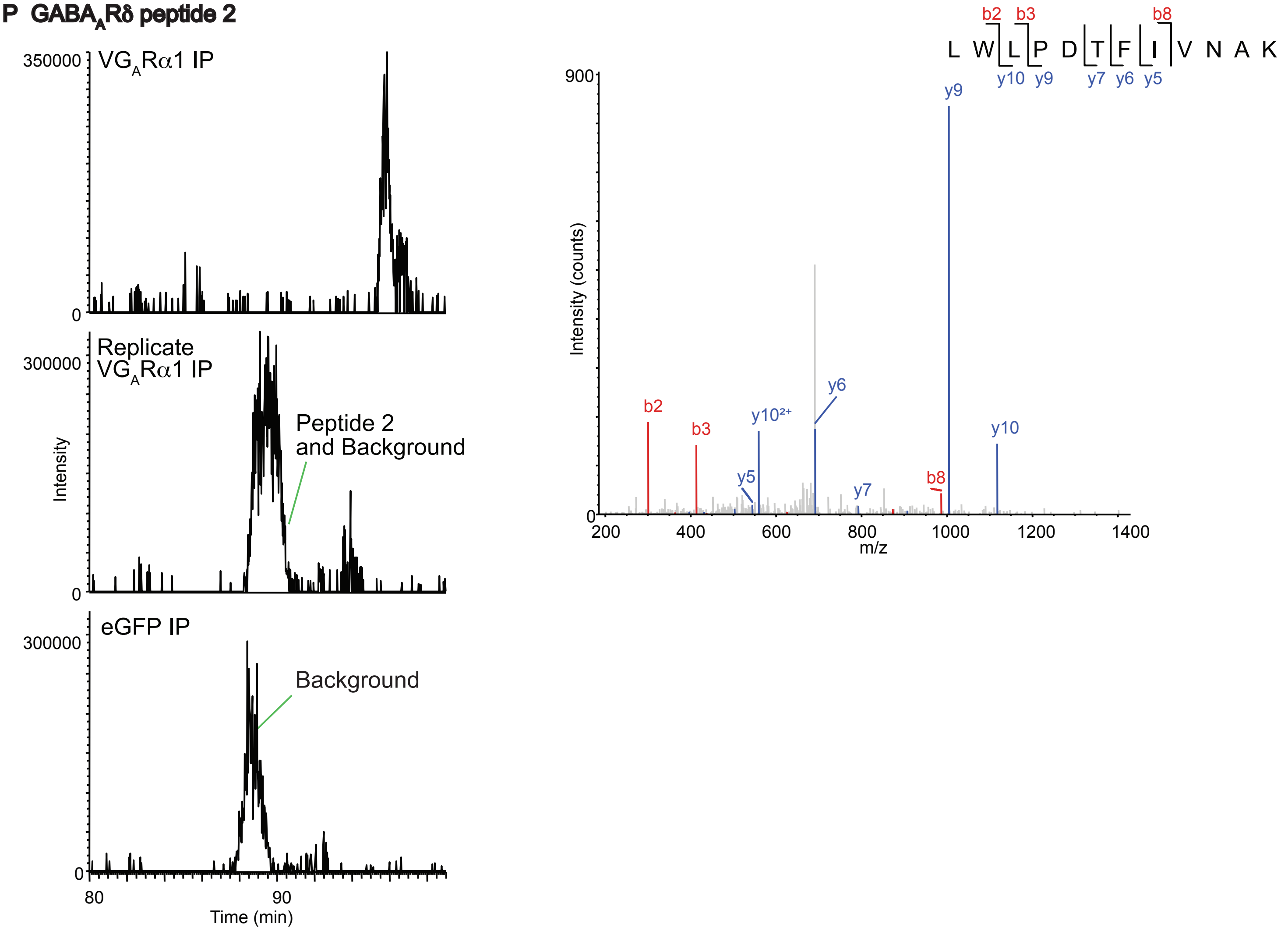

Q Collybistin peptide 1

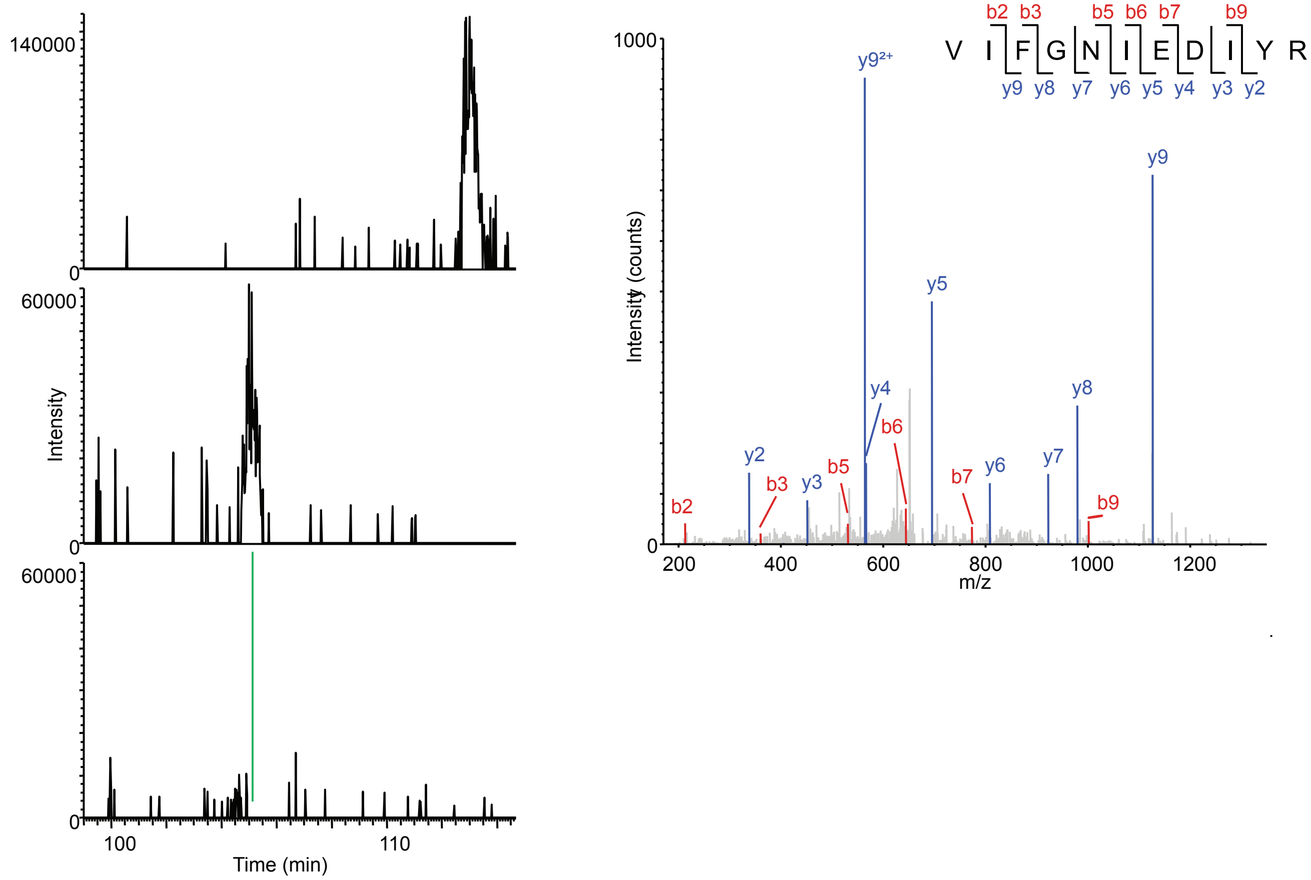

Supplement: Figure S2 — Mass spectrometry identifies proteins present at tagged inhibitory synapses. (A-Q) All peptides were evaluated individually, for their presence or absence in the sample isolated via VGABAARα1 or eGFP, using information from peptide fragmentation spectrum (MS/MS), peptide mass spectrum (MS – not shown), and peptide retention time in extracted ion chromatogram. An example peptide is shown for each protein listed in Table 1. For cases in which MS/MS data was only available for one of the two data sets (VGABAARα1 or VGABAARα1 Replicate), both chromatograms are shown. In cases in which the control chromatogram (eGFP IP) contained a peak at the equivalent retention time, the corresponding MS (not shown) was analyzed to determine whether the peak contained the equivalent or background peptide. (PDF) [file pone.0039572.s002.pdf]
