# Supplementary material for: The Biochemical Anatomy of Cortical Inhibitory Synapses
Source: PLoS One. 2012 Jun 29;7(6):e39572. doi: 10.1371/journal.pone.0039572 (PMC3387162; doi:10.1371/journal.pone.0039572)
Supplement: Table S1 — Detailed analysis of peptides identified by LC-MS/MS. (A) Proteins were identified by the GPM protein sequence database search program X!Tandem using data from the LC/MS/MS experiments. Ensemble ID is the protein accession number in the Ensemble Mouse database. Paralogues sharing some of the indicated peptides are given in brackets. (B) Peptide sequences observed in the LC/MS/MS experiment. The symbol “*” indicates peptide sequences that also appear in protein paralogues. In cases where the peptides are modified, italic Q stands for Pyroglutamate formed at N-terminal Gln, italic M for oxidized Met, italic W for oxidized Trp and italic A for acetylated N terminal Ala. Representative mass chromatograms and MS/MS data for bold format peptides are show in Figure 4 and Figure S3. (C) Experimental peptide molecular masses. (D) Experimental peptide molecular masses – calculated peptide molecular masses. (E) Charge state of the observed peptide ions. (F) Mass to charge ratio of the observed peptide ions. (G) Experimental evidence for peptides’ presence in VGABAARα1 sample. MSMS stands for the peptide being identified via its fragmentation spectrum. MS stands for evidence of the presence of a peptide via its accurately measured mass. (H) Experimental evidence for peptides’ presence in replicate VGABAARα1 sample and GFP control sample. The symbol ‘-’ indicates non-observation of the peptide. (PDF) [file pone.0039572.s003.pdf]

Table S1: Summary of experimental evidences of identified peptides.

(A) Protein name & Ensembl ID	(B) Peptide sequence	(C) Mr	(D) Error	(E) Z	(F) m/z	(G) VG$_A$Rα1 Data 1	(H) VG$_A$Rα1 Data 2	(H) GFP Data 2
GABA$_A$Rβ2	25-QSVNDPSNMSLVK-37	1400.670	0.01	2	701.343	MS/MS	MS/MS	-
ENSMUSP00000007797	111-VADQLWVPDTYFLNDK-126 (*)	1922.952	0.011	2	962.484	MS/MS	MS/MS	-
(GABA$_A$Rβ1, GABA$_A$Rβ3)	111-VADQLWVPDTYFLNDKK-127 (*)	2051.048	0.012	3	684.691	MS/MS	MS/MS	-
	128-SFVHGVTVK-136 (*)	972.543	0.004	2	487.279	MS/MS	MS	-
	142-LHPDGTVLYGLR-153 (*)	1339.732	0.008	3	447.585	MS/MS	MS/MS	-
	154-ITTTAACMMDLR-165 (*)	1382.643	0.008	2	692.330	MS/MS	MS/MS	-
	182-GYTTDDIEFYWR-193 (*)	1564.689	0.006	2	783.352	MS/MS	-	-
	184-TTDDIEFYWR-193 (*)	1344.598	0	2	673.307	MS/MS	-	-
	**194-GDDNAVTGVTK-204**	1075.519	0.005	2	538.767	MS/MS	MS/MS	-
	205-IELPQFSIVDYK-216 (*)	1450.780	0.009	2	726.398	MS/MS	MS/MS	MS
	222-VVFSTGSYPR-231	1111.572	0.007	2	556.794	MS/MS	MS/MS	-
	360-*M*DPHENILLSTLEIK-374	1767.917	0.01	3	590.314	MS/MS	MS/MS	-
	375-NEMATSEAVMGLGDPR-390	1676.762	0.013	2	839.389	MS/MS	MS/MS	-
	391-ST*M*LAYDASSIQYR-404	1620.755	0.01	2	811.385	MS/MS	MS/MS	-
	438-ITIPDLTDVNAIDR-451	1554.834	0.01	2	778.425	MS/MS	MS/MS	MS
GABA$_A$Rβ1	**52-LRPDFGGPPVDVG*M*R-66**	1627.823	0.01	3	543.616	MS/MS	MS/MS	-
ENSMUSP00000031122	112-VADQLWVPDTYFLNDK-127 (*)	1922.952	0.011	2	962.484	MS/MS	MS/MS	-
(GABA$_A$Rβ2, GABA$_A$Rβ3)	112-VADQLWVPDTYFLNDKK-128 (*)	1922.952	0.012	3	641.992	MS/MS	MS/MS	-
	129-SFVHGVTVK-137 (*)	972.543	0.004	2	487.279	MS/MS	MS	-
	143-LHPDGTVLYG LR-154 (*)	1339.732	0.008	3	447.585	MS/MS	MS/MS	-
	155-ITTTAACMMDLR-166 (*)	1382.643	0.008	2	692.330	MS/MS	MS/MS	-
	206-IELPQFSIVDYK-217 (*)	1450.780	0.009	2	726.398	MS/MS	MS/MS	MS
	222-KVEFTTGAYPR-232	1267.660	0.004	2	634.838	MS/MS	MS	-
	359-VQVDAHGNILLSTLEIR-375	1877.042	0.006	2	939.529	MS/MS	-	-
	375-NETSGSEVLTGVSDPK-391	1618.778	0.01	2	810.397	MS/MS	MS/MS	-
	392-AT*M*YSYDSASIQYR-405	1670.736	0.012	2	836.376	MS/MS	MS/MS	-
	396-SYDSASIQYR-405	1188.552	0.012	2	595.284	MS/MS	MS/MS	-
	438-VKIPDLTDVNSIDK-451	1555.852	0.007	3	519.625	MS/MS	MS/MS	-
	440-IPDLTDVNSIDKWSR-454	1757.904	0.01	3	586.976	MS/MS	MS/MS	-
	440-IPDLTDVNSIDK-451	1328.693	0.011	2	665.354	MS/MS	MS	MS
GABA$_A$Rβ3	26-QSVNDPGNMSFVK-38	1404.640	0.006	2	703.328	MS/MS	MS/MS	-
ENSMUSP00000038051	112-VADQLWVPDTYFLNDK-127 (*)	1922.952	0.011	2	962.484	MS/MS	MS/MS	-
(GABA$_A$Rβ1, GABA$_A$Rβ2)	112-VADQLWVPDTYFLNDKK-128 (*)	2051.048	0.012	3	684.691	MS/MS	MS/MS	-
	129-SFVHGVTVK-137 (*)	972.543	0.004	2	487.279	MS/MS	MS	-
	143-LHPDGTVLYGLR-154 (*)	1339.732	0.008	3	447.585	MS/MS	MS/MS	-
	155-ITTTAACMMDLR-166 (*)	1382.643	0.008	2	692.330	MS/MS	MS/MS	-

	183-GYTTDDIEFYWR-194 (*)	1564.689	0.006	2	783.352	MS/MS	-	-
	185-TTDDIEFYWR-194 (*)	1344.598	0	2	673.307	MS/MS	-	-
	206-IELPQFSIVEHR-217	1466.795	0.007	2	734.405	MS/MS	MS	-
	222-NVVFATGAYPR-232	1193.626	0.008	2	597.821	MS/MS	MS/MS	-
	390-NSAISFDNSGIQYR-403	1570.750	0.013	2	786.383	MS/MS	MS/MS	-
	**437-IKIPDLTDVNAIDR-450**	1581.881	0.009	3	528.302	MS/MS	MS/MS	-
Neuroligin-2 ENSMUSP00000053097 (Neuroligin-1)	17-GGGGPGGGAPGGPGLGLGSLGEER-40	1961.965	0.01	2	981.990	MS/MS	MS/MS	-
	57-ELNNEILGPVVQF-69	1470.783	0.012	2	736.399	MS/MS	MS	-
	84-FQPPEAPASWPGVR-97	1537.778	0.011	3	513.601	MS/MS	MS/MS	-
	160-DEATLNPPDTDIR-172	1455.692	0.008	2	728.854	MS/MS	MS	-
	231-GNYGLLDQIQALR-243 (*)	1459.787	0.009	2	730.901	MS/MS	MS/MS	-
	244-WLSENIAHFGGDPER-258	1726.818	0.012	2	864.417	MS/MS	-	-
	315-VGCDREDSTEAVECLR-330	1894.826	0.012	2	948.421	MS/MS	MS	-
	433-FMYTDWADR-441 (*)	1203.507	0.006	2	602.761	MS/MS	MS	-
	450-TLLALFTDHQWVAPAVATAK-469	2152.176	0.009	3	718.400	MS/MS	-	-
	470-LHADYQSPVYFYTFY-484	1912.868	0.002	2	957.442	MS/MS	-	-
	**544-TGDPNQPVPQDTK-556**	1395.668	0.005	2	698.842	MS/MS	MS/MS	-
	557-FIHTKPNR-564	1011.565	0.004	3	338.196	MS/MS	-	-
	565-FEEVVWSK-572	1022.515	0.008	2	512.265	MS/MS	MS/MS	-
	736-ELPPEEELVSLQLK-749	1622.887	0.011	2	812.451	MS/MS	MS/MS	-
	751-GGGVGADPAEALRPACPPDYTLALR-775	2523.267	0.014	3	842.097	MS/MS	MS	-
$GABA_AR\alpha1$ ENSMUSP00000020707 ($GABA_AR\alpha2$, $GABA_AR\alpha3$, $GABA_AR\alpha4$, $GABA_AR\alpha5$)	56-LRPGLGER-63 (*)	896.524	0.005	2	449.270	MS/MS	MS/MS	-
	120-IWTPDTFFHNGK-131 (*)	1461.716	0.013	2	731.866	MS/MS	MS/MS	-
	147-ITEDGTLLYTMR-158	1411.709	0.008	2	706.863	MS/MS	MS/MS	-
	163-AECP*M*HLEDFP*M*DAHACPLK-182	2400.022	0.022	3	801.015	MS/MS	MS	-
	163-AECPMHLEDFPMDAH-177 (*)	1798.718	0.007	3	600.580	MS/MS	MS	-
	191-AEVVYEWTR-199	1151.567	0.006	2	576.791	MS/MS	MS/MS	MS
	204-SVVVAEDGSR-213 (*)	1017.513	0.004	2	509.764	MS/MS	MS/MS	-
	339-RGYAWDGK-346	951.460	0.005	2	476.738	MS/MS	MS/MS	-
	**340-GYAWDGK-346**	795.359	0.004	2	398.687	MS/MS	MS/MS	-
	363-K*N*NTYAPTATSYTPNLAR-380	1982.978	0.008	3	662.000	MS/MS	MS/MS	-
	363-KNNTYAPTATSYTPN-377	1641.771	0.008	2	821.894	MS/MS	MS/MS	-
	364-*N*NTYAPTATSYTPNLAR-380	1854.888	0.014	2	928.452	MS/MS	MS/MS	-
	398-EVKPETKPPEPK-409	1377.755	0.005	2	689.885	MS/MS	MS/MS	-
Gephyrin ENSMUSP00000054064	2-*A*TEG*M*ILTNHDHQIR-16	1792.863	0.011	3	598.629	MS/MS	MS	-
	41-DLVQDPSLLGGTISAYK-57	1775.936	0.006	2	888.976	MS/MS	MS/MS	MS

	78-ELNLILTTGGTGFAPR-93	1658.912	0.014	2	830.464	MS/MS	MS	-
	139-TLIINLPGSK-148	1054.643	0.005	2	528.330	MS/MS	MS/MS	MS
	244-IPDSIISR-251	899.513	0.006	2	450.764	MS/MS	MS/MS	MS
	279-LSTASCPTPK-288	1060.527	0.005	2	531.271	MS/MS	MS/MS	-
	335-ASHSAVDITK-344	1027.536	0.007	3	343.520	MS/MS	MS	-
	351-*M*SPFPLTSMDK-361	1268.589	0.012	2	635.302	MS/MS	-	-
	387-VLAQDVYAK-395	1005.554	0.005	2	503.785	MS/MS	MS	-
	396-DNLPPFPASVK-406	1183.630	0.007	2	592.823	MS/MS	MS/MS	-
	421-FIIGESQAGEQPTQTVMPGQVMR-443	2503.227	0.007	3	835.417	MS/MS	-	-
	**468-ESDDGTEELEVR-479**	1377.589	0	2	689.802	MS/MS	MS/MS	-
$GABA_AR\gamma2$ ENSMUSP00000063812 ($GABA_AR\gamma1$)	60-VPEGDVTVILNNLLEGYDNK-79	2201.132	0.011	2	1101.574	MS/MS	-	-
	80-LRPDIGVK-87	896.551	0.007	2	449.283	MS/MS	MS	-
	144-IWIPDTFFR-152 (*)	1193.630	0.006	2	597.823	MS/MS	MSMS	-
	156-KADAHWITTPNR-167	1408.729	0.008	3	470.584	MS/MS	MS/MS	MS
	202-SCPLEFSSYGYPR-214	1561.696	0.01	2	781.856	MS/MS	MS	-
	208-SSYGYPR-214	828.378	0.002	2	415.197	MS/MS	MS	-
	224-SSVEVGDTR-232	948.455	0.005	2	475.236	MS/MS	MS/MS	-
	236-LYQFSFVGLR-245	1228.665	0.005	2	615.340	MS/MS	MS/MS	-
	393-SATIQMNNATHLQER-407	1712.832	0.006	2	857.424	MS/MS	MS	-
	**408-DEEYGYECLDGK-419**	1476.581	0.01	2	739.298	MS/MS	MS/MS	-
$GABA_AR\alpha3$ ENSMUSP00000062638 ($GABA_AR\alpha1$, $GABA_AR\alpha2$, $GABA_AR\alpha4$, $GABA_AR\alpha5$)	**34-RQEPGDFVK-42**	1074.551	0.006	2	538.283	MS	MS/MS	-
	146-IWTPDTFFHNGK-157 (*)	1461.703	0.013	2	731.859	MS/MS	MS/MS	-
	240-LNQYDLLGHVVGTEIIR-256	1939.052	0.017	3	647.359	MS/MS	-	-
	409-DTEFSTISK-417	1026.486	0.011	2	514.251	MS/MS	MS	-
	418-SAAAPSASSTPTAIASPK-435	1613.825	0.014	2	807.920	MS/MS	MS	MS
	436-ATYVQDSPAETK-447	1308.619	0.01	2	655.317	MS/MS	MS	-
Neuroligin-3 ENSMUSP00000066304 (Neuroligin-2)	231-GNYGLLDQIQALR-243 (*)	1459.787	0.009	2	730.901	MS/MS	MS/MS	-
	315-VGCNVLDTVDMVDCLR-330	1864.857	0.009	2	933.436	MS/MS	-	-
	433-FMYTDWADR-441 (*)	1203.507	0.006	2	602.761	MS/MS	MS	-
	544-TGDPNKPVPQDTK-556	1395.705	0.007	3	466.243	MS/MS	MS	-
	**763-LTALPDYTLTLR-774**	1375.780	0.009	2	688.898	MS/MS	MS/MS	MS
Neurobeachin ENSMUSP00000029374	559-AVLEQFLSFAK-569	1251.697	0.011	2	626.856	MS/MS	-	-
	1293-VDLGFR-1298	705.383	0.002	2	353.699	MS/MS	MS	-
	1891-EIFVDFAPFLSR-1902	1439.755	0.011	2	720.885	MS/MS	-	-
	1945-NAGLAFIELINEGR-1958	1515.818	0.014	2	758.917	MS/MS	-	-
	**2087-SAVEYGTEEDVVK-2099**	1424.678	0.012	2	713.347	MS/MS	MS	-
	2247-VGVGTSYGLPQAR-2259	1303.688	0.008	2	652.852	MS/MS	MS	-
$GABA_AR\alpha2$ ENSMUSP00000000572 ($GABA_AR\alpha1$, $GABA_AR\alpha3$,	121-IWTPDTFFHNGK-132 (*)	1461.703	0.013	2	731.859	MS/MS	MS/MS	-
	148-IQDDGTLLYTMR-159	1424.705	0.009	2	713.360	MS/MS	MS/MS	-
	164-AECPMHLEDFPMDAH-178 (*)	1798.718	0.007	3	600.580	MS/MS	MS	-
	**215-LNQYDLLGQSIGK-227**	1447.777	0.011	2	724.896	MS/MS	MS/MS	-

GABA$_A$Rα4, GABA$_A$Rα5)	390-SATTPEPNKKPENKPAEAK-408	2036.073	0.02	3	679.699	MS/MS	MS	-
GABA$_A$Rα4 ENSMUSP00000031121 (GABA$_A$Rα1, GABA$_A$Rα2, GABA$_A$Rα3, GABA$_A$Rα5)	**51-ILDSLLDGYDNR-62**	1392.698	0.01	2	697.357	MS/MS	MS/MS	-
	190-FGSYAYPK-197	931.444	0.001	2	466.730	MS/MS	MS	-
	355-ISKPPPEVPAAPVLK-369	1541.930	0.013	3	514.985	MS/MS	MS	-
	390-TNALVHSESDVK-401	1298.657	0.011	2	650.336	MS/MS	MS	-
	413-TSAVQESSEATPK-425	1333.640	0.005	2	667.828	MS/MS	MS	-
GABA$_A$Rα5 ENSMUSP00000063276 (GABA$_A$Rα1, GABA$_A$Rα2, GABA$_A$Rα3, GABA$_A$Rα4)	**52-ILDGLLDGYDNR-63**	1362.686	0.008	2	682.351	MS	MS/MS	-
	64-LRPGLGER-71 (*)	896.524	0.005	2	449.270	MS/MS	MS/MS	-
	128-IWTPDTFFHNGK-139 (*)	1461.716	0.013	2	731.866	MS/MS	MS/MS	-
	212-SVVVAEDGSR-221 (*)	1017.513	0.004	2	509.764	MS/MS	MS/MS	-
	392-EQPPAGTANAPTVSIK-407	1579.827	0.007	2	790.921	MS/MS	MS	-
Neurexin 1 ENSMUSP00000057294	173-TGSISFDFR-181	1028.499	0.007	2	515.257	MS/MS	-	-
	**824-LAIGFSTVQK-833**	1062.614	0.007	2	532.315	MS/MS	MS	MS
	1084-EPYPGSAEVIR-1094	1216.616	0.008	2	609.316	MS/MS	-	-
	1126-NRDEGSYHVDESR-1138	1562.679	0.008	3	521.901	MS/MS	-	-
GABA$_A$Rγ1 ENSMUSP00000031119 (GABA$_A$Rγ2)	59-IHEGDITQILNSLLQGYDNK-78	2270.164	0.01	3	757.729	MS/MS	-	-
	79-LRPDIGVR-86	924.556	0.006	3	309.193	MS/MS	MS	-
	143-IWIPDTFFR-151 (*)	1193.630	0.006	2	597.823	MS/MS	MSMS	-
	**155-KSDAHWITTPNR-166**	1424.726	0.011	3	475.917	MS/MS	MS	-
	235-LYQFAFVGLR-244	1212.665	0	2	607.340	MS/MS	MS	-
Lipoma HMGIC fusion partner-like 4 protein ENSMUSP00000061172	10-LYHEHYMR-17	1147.527	0.004	2	574.771	MS/MS	MS	MS
	**79-GSFTDFSTIP SSAFK -93**	1590.764	0.01	2	796.390	MS	MSMS	-
	166-YSLGDCSVR-174	1055.473	0.003	2	528.744	MS/MS	MS	-
	201-QTDLLQEELK-210	1215.640	0.006	2	608.828	MS/MS	MS	-
	215-DFVGTTVSSVLRPGGDVSGWGVLPCPVAHTQGP-247	3348.676	0.021	3	1117.233	MS/MS	-	-
GABA$_A$Rδ ENSMUSP00000030925	119-LWLPDTFIVNAK-130	1415.789	0.007	2	708.902	MS/MS	-	-
	**416-LKPIDADTIDIYAR-429**	1602.870	0.009	3	535.297	MS/MS	MS	-
Collybistin ENSMUSP00000085403	**154-VIFGNIEDIYR-164**	1337.709	0.012	2	669.862	MS/MS	MS	-
