# Supplementary material for: The Biochemical Anatomy of Cortical Inhibitory Synapses
Source: PLoS One. 2012 Jun 29;7(6):e39572. doi: 10.1371/journal.pone.0039572 (PMC3387162; doi:10.1371/journal.pone.0039572)
Supplement: Text S1 — Detailed experimental procedures and supporting information references. (DOCX) [file pone.0039572.s004.docx]

**SUPPORTING INFORMATION TEXT S1**

**DETAILED EXPERIMENTAL PROCEDURES**

**Two-Electrode Voltage-Clamp Recordings of Xenopus Oocytes**

The cDNA encoding *GABRA1* together with the 3’UTR was amplified from cortical RNA, and placed in frame with a preprotrypsin signal sequence and Venus in a building vector based on eGFP-C2 (Clontech, Mountain View, USA). cDNA encoding for the *Venus-GABRA1* subunit was subcloned into the pCS2A plasmid for oocyte expression. cDNA clones of the rat subunits *GABRA1, GABRB1, GABRB2, GABRG2* in pGEMHE plasmid were a generous gift from Dr. Myles Akabas (Albert Einstein Institute, New York). All clones were in vitro transcribed with T7 or SP6 RNA polymerases (mMESSAGE mMACHINE, Ambion, Austin, TX) as described [1]. Oocytes were surgically removed and prepared as described [2]. Each oocyte was injected with 20 nl of a cRNA mix containing various combinations of the subunuits in 1:1 ratio. Macroscopic currents were recorded 2 days after injection with a GeneClamp 500B amplifier (Axon Instruments) using a two-electrode voltage clamp with active ground configuration. Electrodes (0.5–2 MΩ) were filled with 3M KCl. The extracellular recording solution contained 82.5 mM NaCl, 2 mM KCl, 1 mM CaCl2, 1 mM MgCl2, and 10 mM HEPES at pH 7.4. g - aminobutyric acid (GABA) (Sigma-Aldrich) was prepared in extracellular solution at concentration of 10µM. Solutions were gravity fed with a flow rate of 5 ml/min using a Bath Perfusion System valve controller (ALA-VM8, ALA Scientific Instruments). Data were acquired using pCLAMP9 software (Axon Instruments) and currents were sampled at 10 Hz. Membrane potential was clamped to 70 mV; only oocytes with leak currents <100 nA were used. Current amplitude was evaluated by dividing peak amplitudes of 5 single oocytes. All experiments were repeated twice. Results are presented as means ± SEM.

**Lentivirus Production**

Recombinant lentiviral vectors were prepared using transient transfection of HEK293T cells as described [3]. Briefly, 5x10^6^ HEK293T cells were seeded on 24 10 cm cell-culture dishes precoated with poly-L-lysine (Sigma-Aldrich). The next day, the transfer vector plus the packaging vectors pLP1 and pLP2 (ViraPower System; Invitrogen) and the envelope vector pCMV-VSV-G (Addgene) were cotransfected with PEI transfection (Polyethylenimine MAX, Polysciences Inc.) into HEK293T cells. The transfection mix was prepared as follows (for one dish): 12 mg transfer plasmid, 6.5 mg pLP1, 3.5 mg pLP2, and 3.5 mg pCMV-VSV-G were added to 800 ml OptiMEM (GIBCO) and incubated at RT for 5 min. In the meanwhile, 51 ml PEI solution (1 mg/ml) was added to 800 ml OptiMEM in a separate tube. Both solutions were mixed and incubated at RT for 30 min. During the incubation time, the medium of the cells was changed to 5 ml OptiMEM per dish. Finally, 3 ml transfection mix was added to each dish. After 7–8 hr, the medium was replaced by 9 ml OptiMEM per dish, without addition of FBS. The virus-containing medium was harvested 40–45 hr after medium change, cleared by centrifugation at 2500x g for 10 min at 4°C, and filtered through 0.45 mm filter units (Millipore). Virus concentration was carried out by ultracentrifugation in a Beckman Optima L-90K ultracentrifuge using a SW32 Ti rotor at 50,000xg for 3 hr with a purification layer of 1 ml 20% sucrose (in dH20) added to the bottom of the centrifuge tubes. Subsequently, the virus was resuspended in 50–100 ml 1x PBS, aliquoted, and stored at -70°C. The titer of concentrated lentivirus was determined by transducing 1x105 HEK293T cells per well in a 24-well cell-culture plate with limiting virus dilutions and quantification of GFP-positive cells by FACS analysis after 3 days. Titers of concentrated lentivirus were in the range of 5x10^8^ – 2 x10^9^ TU ml^–1^.

**Primary neuronal culture.**

Dissociated hippocampal cultures were prepared from embryonic day 19 rat embryos and prepared as described [3]. Briefly, pregnant Wistar-Han rats were anesthetized and embryos were dissected in ice-cold dissection buffer (HBSS with 25 mM glucose and 15 mM HEPES). Hippocampi were isolated and after digestion (1 mg trypsin in 5 ml of 1× PBS with 20 mM glucose and 15 mM HEPES) for 8 min at 37 °C, the reaction was stopped by replacing the digestion solution with trypsin inhibitor solution (15 mg trypsin inhibitor; Invitrogen) in 13 ml of Neurobasal medium (Invitrogen). Subsequently, the tissue pellet was resuspended in 2 ml culture medium (95% neurobasal medium, 1% FBS, 2% B27 supplement, 1% antibiotic-antimycotic solution, 0.5 mM [Glutamax (Invitrogen)](http://www.biocompare.com/natureproducts/go.asp?id=nmeth.1425_p_p8), 10 μM L-glutamate, 25 μM β- [mercaptoethanol (Sigma-Aldrich)](http://www.biocompare.com/natureproducts/go.asp?id=nmeth.1425_p_p9) and the cells were triturated by pipetting up and down 10–15 times with a 1 ml pipette. The upper 1.5 ml of the cell suspension was then transferred to a new tube. The cells were seeded in a density of 2.5 × 10^5^ cells per well on poly(D-/L- [ornithine](http://www.biocompare.com/natureproducts/go.asp?id=nmeth.1425_p_p10)) (Sigma-Aldrich) coated glass coverslips (13 mm) in a 24-well plate in culture medium. Cells were incubated at 37 °C and 5% CO_2_, and one-third of the medium was replaced twice a week, starting at 5 d *in vitro* with culture medium without FBS and glutamate.

**Animals**

The sequence encoding *VGABRA1* and the SV40 polyadenylation signal from the building vector were subcloned into the PL53.SC-AB shuttle vector. The Otx1 containing BAC RP23-106C14 was modified by homologous recombination using this shuttle vector and the two-step method [4]. Recombination boxes of 1kb were amplified from the BAC genomic DNA using the following primers: for box A, 5’AGCTTTGTTTAAACGCTAACAGCCGGGTGGAGGT3’ and 5’TTGGCGCGCCGGCCTTCCAAAATCCCTAGA3’; for box B, 5’AAGGAAAAAAGCGGCCGCCTGAGGGGACATGCGAGA3’ and 5’CGACGCGTACCTCAAACAACCCCCATAC3’. Injection of the BAC into the mouse genome was performed and verified based on published protocols [4]. Otx1-eGFP mice were obtained from GENSAT and generated as described [4]. BAC transgenic mice were bred on the FVB background and littermates were used as wild-type controls. All experiments using animals were performed according to protocols approved by the Institutional Animal Care and Use Committee at The Rockefeller University.

**Immunofluorescence and Confocal Laser Scanning Microscopy**

For images taken at lower magnification sections were prepared according to previously published protocols [5]. Mice were transcardially perfused with ice-cold phosphate buffered saline (PBS), followed by 4% paraformaldehyde in PBS, postfixed for 12 h in the same solution and equilibrated in 30% sucrose. Serial free-floating coronal sections (35 μm) through the cortex were processed for immunofluorescence. For images taken at higher magnification, mice were euthanized by CO_2_ asphyxiation and fresh frozen sections (14 μm) were prepared using a cryostat. Slide-mounted sections were fixed for 30 sec in 4% paraformaldehyde in PBS, washed in PBS and double or triple immunofluorescence staining was performed [6]. The following primary antibodies were used: chicken anti-eGFP (1:2000, ab13970, Abcam, Cambridge, MA), rabbit anti-PSD95 (1:500, ab18258, Abcam, Cambridge, MA), mouse anti-GABA_A_ receptor β2/3, clone BD17 (1:100, MAB341, Millipore, Billerica, MA), mouse anti-Gephyrin (1:700, 147011, Synaptic Systems, Goettingen, Germany), rabbit anti-Nlgn2 (1:2000), and rabbit anti-Nlgn3 (1:1000, Neuroligin antibodies were a generous gift from Peter Scheiffele). All secondary antibodies were raised in goat and conjugated to Alexa-Fluor488, 568 or 647 (Invitrogen). The sections were analyzed by confocal laser scanning microscopy (Zeiss LSM 510) using sequential acquisition of separate wavelength channels to avoid fluorescent bleed through. Stacks of 20-50 confocal images were taken using a 40x or 100x objective and analyzed using Image J software (National Institutes of Health).

**Immuno-Electron Microscopy**

Double immuno-electron microscopy was performed to determine whether Venus-GABA_A_Rα1 occur postsynaptic to GABAergic axon terminals, latter of which were identified by the presence of the GABA-synthesizing enzyme, glutamic acid decarboxylase (GAD). Double labeling used 3,3-diaminobenzidine HCl (DAB) and silver-intensiﬁed colloidal gold (SIG) as immunolabels [7]. Brains of WT and Otx1-VGABA_A_Rα1 transgenic mice were fixed by transcardial perfusion of 0.1 % glutaraldehyde, mixed with 4.0% paraformaldehyde in 0.1 M phosphate buffer (PB, pH 7.4). Prior to immunohistochemistry, tissue was permeabilized by cryoprotection in dimethylsulfoxide followed by five to six cycles of freeze-thawing, using liquid nitrogen-cooled isopentane. Cortical sections were incubated in PBS- bovine serum albumin (BSA) (Sigma Chemicals, Saint Louis, MO)-azide with 0.06% Triton X-100 containing primary antibodies, goat anti-GFP (1:500) or rabbit anti-GFP (1/500, ab6556, Abcam, Cambridge, MA) to recognize Venus-GABA_A_α1, and rabbit anti-GAD65/67 (1:400, AB1511, Millipore, Billerica, MA). The goat anti-GFP antiserum was generated by injection in a goat of the full-length GFP (Green Mountain Antibodies, Burlington, VT) and was affinity-purified using a column made of Sepharose-4B resin coupled to full-length GFP. For immunolabeling with DAB, sections were incubated in biotinylated donkey anti-rabbit or donkey anti-goat IgG (15 mg/ml, Vector Laboratories, Burlingame) followed by incubation in the ABC solution (Elite Kit, Vector Laboratories, Burlingame). For immunolabeling with SIG, sections were incubated for 16 hour in ultrasmall (0.8 nm) gold-conjugated donkey anti-goat IgG or donkey anti-rabbit IgG (Electron Microscopy Sciences, Washington) at a dilution of 1:100 in PBS-BSA (pH 7.6). Sections were then postﬁxed in 1.0% glutaraldehyde and rinsed for 1 min in 0.2 M citrate buffer (pH 7.4) prior to immersion in silver intensiﬁcation reagent (Silver Enhancer Kit, KPL, Maryland). Multiple postﬁxation steps were employed to preserve ultrastructure: 1.0% glutaraldehyde with PBS (pH 7.4) for 10 min; 0.1% osmium tetroxide (in 0.1 M PB) for 30 minutes; and 1.0% uranyl acetate in 70% ethanol, overnight. Control experiments were performed and did not detect nonspecific labeling nor cross reactivity by the indicated secondary antibodies.

Layer 5/6 pyramidal neurons were identified by both position and immunoreactivity for the transgene. Images used for the Figure were captured digitally using a Hamamatsu CCD camera attached to a JEOL 1200XL electron microscope at a magnification of 40,000x and spanning an area of 29 mm^2^.

**Preparation of Synaptic Protein Complexes and Affinity Purification**

Five cortices from adult mice were used for the preparation of a crude synaptosome fraction based on previously published protocols [8]. The solution used as a homogenization and resuspension buffer contained 0.32 M sucrose, 5mM HEPES, 0.1 mM EDTA, pH=7.3 and a protease inhibitor cocktail (Sigma, Saint Louis). P2 was then solubilized 30 minutes at 4°C using a final concentration of 0.1% Triton X-100. The cleared solubilized fraction was separated by gravity flow on a gel-filtration column (Sephacryl S1000 Superfine, GE Healthcare) prepared using a solution containing 2 mM CaCl_2_, 132 mM NaCl, 3 mM KCl, 2 mM MgSO_4,_ 1.2 mM NaH_2_PO_4_, 10 mM HEPES and 0.1% Triton X-100, pH=7.4. 2 ml fractions were collected and aliquots were used for protein dosage using the BCA Protein assay kit (Pierce, Rockford). Calibration of the gel-filtration column was performed using the gel filtration HMW calibration kit (GE Healthcare).

Pooled fractions from the column were used for affinity-purification of tagged inhibitory synaptic protein complexes. Dynabeads Protein G beads (Dynal, Oslo) were conjugated in 0.15 M KCl for 2 hours at room temperature using 0.88 mg of mouse monoclonal anti-eGFP antibody per 1mL of beads. Following conjugation, the antibody was crosslinked to Protein G with 20 mM Dimethyl pimelimidate•2 HCl (Pierce, Rockford, IL) in 0.2 M triethanolamine, pH 8.0. The crosslinking reaction was stopped with 50 mM Tris pH 8.0. 5mL of beads were used for affinity-purification of pooled synaptic fractions from 5 cortices during forty-five minutes at 4°C. Beads were then washed in 2 mM CaCl_2_, 300 mM NaCl, 3 mM KCl, 2 mM MgSO_4,_ 1.2 mM NaH_2_PO_4_, 10 mM HEPES and 0.1% Triton X-100. Purified complexes were eluted in 1.0 N NH_4_OH, 0.5 mM EDTA. Biochemical preparations and affinity-purifications were performed in parallel for each genotype. The mouse monoclonal anti-eGFP antibody using for immunopurification (clone 19F7) was generated by immunizing mice with purified GST-eGFP fusion protein (Monoclonal Antibody Core Facility at Memorial Sloan-Kettering Cancer Center, New York) [9].

**Mass Spectrometry**

Mass spectrometry analysis was performed on proteins affinity isolated via VGABA_A_Rα1 (tagged sample) or eGFP (control sample) and digested using the “FASP II” on-membrane digestion protocol [10] using an LTQ-Orbitrap XL mass spectrometer. After LC-MS/MS data were acquired for the control sample, a peak list of m/z values was generated for sequenced peptides and used to construct a “reject mass list” (with retention time windows of ±4 min) during data acquisition for the tagged protein sample. The resulting LC-MS/MS data were utilized to identify proteins present in the control sample as well as additional proteins that were only present in the tagged protein sample. Protein identification was carried out by using The Global Proteome Machine (GPM) database search program X!Tandem (http://ppp.thegpm.org/tandem/ppp.html) [11] to yield a highly specific cohort of inhibitory synaptic proteins with an expectation value cutoff score of E = 10^-4^. This confidence level was independently confirmed using the target decoy strategy [12]. Proteins that were uniquely affinity isolated via VGABA_A_Rα1 were then determined by subtracting proteins that were immunoisolated via eGFP. Table S1 shows all identified peptides of the inhibitory synaptic proteins that were either unique to the fusion protein sample or highly enriched over the control.

**SUPPORTING INFORMATION REFERENCES**

1. Ibañez-Tallon I, Pagenstecher A, Fliegauf M, Olbrich H, Kispert A, et al. (2004) Dysfunction of axonemal dynein heavy chain Mdnah5 inhibits ependymal flow and reveals a novel mechanism for hydrocephalus formation. Hum Mol Genet 13: 2133-2141.

2. Stürzebecher AS, Hu J, Smith ESJ, Frahm S, Santos-Torres J, et al. (2010) An in vivo tethered toxin approach for the cell-autonomous inactivation of voltage-gated sodium channel currents in nociceptors. J Physiol (Lond) 588: 1695-1707.

3. Auer S, Stürzebecher AS, Jüttner R, Santos-Torres J, Hanack C, et al. (2010) Silencing neurotransmission with membrane-tethered toxins. Nat Meth 7: 229-236.

4. Gong S, Yang X, Li C, Heintz N (2002) Highly efficient modification of bacterial artificial chromosomes (BACs) using novel shuttle vectors containing the R6Kgamma origin of replication. Genome Res 12: 1992-1998.

5. Earnheart JC, Schweizer C, Crestani F, Iwasato T, Itohara S, et al. (2007) GABAergic control of adult hippocampal neurogenesis in relation to behavior indicative of trait anxiety and depression states. J Neurosci 27: 3845-3854.

6. Schneider Gasser EM, Straub CJ, Panzanelli P, Weinmann O, Sassoè-Pognetto M, et al. (2006) Immunofluorescence in brain sections: simultaneous detection of presynaptic and postsynaptic proteins in identified neurons. Nat Protoc 1: 1887-1897.

7. Aoki C, Rodrigues S, Kurose H (2000) Use of electron microscopy in the detection of adrenergic receptors. Methods Mol Biol 126: 535-563.

8. Dunkley P, Heath J, Harrison S, Jarvie P, Glenfield P, et al. (1988) A rapid Percoll gradient procedure for isolation of synaptosomes directly from an S1 fraction: homogeneity and morphology of subcellular fractions. Brain Res 441: 59-71.

9. Heiman M, Schaefer A, Gong S, Peterson JD, Day M, et al. (2008) A translational profiling approach for the molecular characterization of CNS cell types. Cell 135: 738-748.

10. Wiśniewski JR, Zougman A, Nagaraj N, Mann M (2009) Universal sample preparation method for proteome analysis. Nat Meth 6: 1-5.

11. Beavis RC (2006) Using the global proteome machine for protein identification. Methods Mol Biol 328: 217-228.

12. Elias JE, Gygi SP (2007) Target-decoy search strategy for increased confidence in large-scale protein identifications by mass spectrometry. Nat Meth 4: 207-214.
